# Aligning Metabolic Pathways Exploiting Binary Relation of Reactions

**DOI:** 10.1371/journal.pone.0168044

**Published:** 2016-12-09

**Authors:** Yiran Huang, Cheng Zhong, Hai Xiang Lin, Jing Huang

**Affiliations:** 1 School of Computer Science and Engineering, South China University of Technology, Guangzhou, China; 2 School of Computer, Electronics and Information, Guangxi University, Nanning, China; 3 Faculty of Electrical Engineering, Mathematics and Computer Science, Delft University of Technology, Delft, The Netherlands; 4 State Key Laboratory for Conservation and Utilization of Subtropical Agro-bioresources, Guangxi University, Nanning, China; Jilin University, CHINA

## Abstract

Metabolic pathway alignment has been widely used to find one-to-one and/or one-to-many reaction mappings to identify the alternative pathways that have similar functions through different sets of reactions, which has important applications in reconstructing phylogeny and understanding metabolic functions. The existing alignment methods exhaustively search reaction sets, which may become infeasible for large pathways. To address this problem, we present an effective alignment method for accurately extracting reaction mappings between two metabolic pathways. We show that connected relation between reactions can be formalized as binary relation of reactions in metabolic pathways, and the multiplications of zero-one matrices for binary relations of reactions can be accomplished in finite steps. By utilizing the multiplications of zero-one matrices for binary relation of reactions, we efficiently obtain reaction sets in a small number of steps without exhaustive search, and accurately uncover biologically relevant reaction mappings. Furthermore, we introduce a measure of topological similarity of nodes (reactions) by comparing the structural similarity of the *k*-neighborhood subgraphs of the nodes in aligning metabolic pathways. We employ this similarity metric to improve the accuracy of the alignments. The experimental results on the KEGG database show that when compared with other state-of-the-art methods, in most cases, our method obtains better performance in the node correctness and edge correctness, and the number of the edges of the largest common connected subgraph for one-to-one reaction mappings, and the number of correct one-to-many reaction mappings. Our method is scalable in finding more reaction mappings with better biological relevance in large metabolic pathways.

## Introduction

In the last few decades, the quantity and quality of metabolic data in biological databases such as KEGG (Kyoto Encyclopedia of Genes and Genomes) [1] and Metacyc [2] are greatly increased. The comparative analysis of this vast quantity of metabolic data provides insights into biology and life science applications [3]. An effective way of such analysis is to find the similarity between metabolic pathways by aligning them. The similarity between two pathways is often modeled as a function of the similarity between the aligned nodes or matching edges [4]. By comparing the similarity between metabolic pathways, we can reconstruct phylogeny and infer unknown function or evolution of pathways [5], reveal similar connecting pattern of metabolic pathways [6], and study the structural and functional relevance among species [7, 8]. The complexity of the pathway alignment problem stems from its close relationship with graph and subgraph isomorphism problems, which are GI (Graph isomorphism)-Complete and NP-Complete respectively [4]. Thus, it may become impractical to find an accurate solution for this problem as the size of the pathways grows. Due to both computational hardness of pathway alignment and the increasing amount of available metabolic data, obtaining topologically and biologically accurate alignments is a challenging task [9].

In the metabolic pathway alignment problem, metabolic pathways are usually represented as directed graphs, where a node denotes a molecule which can be specified as reaction, enzyme, or compound and an edge represents the interactions between molecules. A one-to-one mapping between nodes in two metabolic pathways maps a node from one pathway to a node in the other. A one-to-many mapping between the nodes in two metabolic pathways maps a node from one pathway to a connected subgraph of many nodes in the other [10]. The size of a one-to-many mapping is determined by the number of nodes in this mapping. Performing an alignment is often considered as finding one-to-one mappings or one-to-many mappings between molecules in metabolic pathways [10].

Accordingly, we can categorize existing literature on metabolic pathway alignment into two types. The first type finds one-to-one mappings between molecules of metabolic pathways to identify similar parts in different pathways [3, 11–20]. This type of methods can be generally classified into two categories: (1) graph-based isomorphism methods. (2) dynamic programming methods.

The graph isomorphism problem asks to decide whether two given graphs are isomorphic, and the subgraph isomorphism problem asks to decide whether one graph is isomorphic to a subgraph of another [11]. A straightforward method for identifying the similarity between metabolic pathways is to transform metabolic pathway alignment problem into graph-based isomorphism problem. Considerable efforts were devoted to aligning metabolic pathways in this way [3, 11–17]. For example, Pinter *et al*. [12] used enzyme graph to describe metabolic pathway and proposed a tree-based pathway search method called MetaPathwayHunter to align the enzyme graphs by using a graph theoretic approach. Although MetaPathwayHunter obtains a high efficiency in the alignments, the pathways are restricted to trees. To alleviate this restriction, Wernicke and Rasche [13] reduced the pathway alignment problem to subgraph homeomorphism problem and presented an alignment tool METAPAT. METAPAT does not restrict the topology of the metabolic networks in the alignments. Given two metabolic networks *G*_*P*_ and *G*_*H*_ where *G*_*P*_ is represented as the pattern network and *G*_*H*_ is represented as the host network, METAPAT determines whether *G*_*H*_ contains a subgraph that is isomorphic to *G*_*P*_ [13]. Owing to the fact that subgraph homeomorphism problem is NP-complete, METAPAT could be computationally hard with the increasing size of the networks. Meanwhile, Yang and Sze [14] proposed two metabolic pathway matching methods PathMatch and GraphMatch. PathMatch reduces the path matching problem to finding the longest weighted path in a directed acyclic graph while GraphMatch reduces the graph matching problem to finding the highest scoring subgraphs in a graph. Both PathMatch and GraphMatch can effectively and accurately extract biologically meaningful pathways, but finding the matching is time consuming owing to the exhaustive search of subgraphs. Although graph-based isomorphism is the most straightforward idea for aligning pathways, the computational complexity of the graph-based isomorphism problem prohibits its practical application because implementation requires tremendous computing resources as the size of the pathways grows.

In addition, some other methods align metabolic pathways by employing dynamic programming [18–20]. In such alignment methods, the similarity between two pathways is defined by the sum of both node and edge matching scores in the similarity objective function. Then, the alignment of pathways is solved by maximizing the similarity objective function between two pathways over all feasible combinations. MNAligner [18] is one example of such methods. MNAligner uses the integer quadratic programming to formulate the alignment of two pathways and find conserved patterns between pathways. To align both two and multiple pathways, Tohsato *et al*. [19] exploited the global alignment algorithm using dynamic programming to find common pattern from pairwise alignment and then extend pairwise alignment to multiple alignment. Tohsato *et al*.’s methods were successfully applied to pathway analyses of sugar, DNA and amino acid metabolisms. However, dynamic programming methods do not work well for the large pathway alignment problem since solving the large-scale dynamic programming is time consuming.

**Fig 1:**
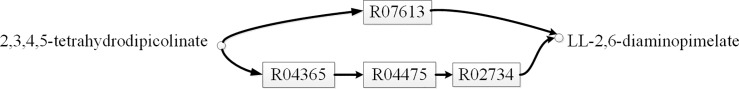
A part of lysine biosynthesis pathway. The square rectangles represent reactions. The compounds are depicted by small circles. Reactions are represented by their KEGG identifiers. Plants use the upper path with a reaction, whereas *H*. *sapiens* (human) accomplishes this transformation through the lower path with three reactions.

Although the above-mentioned methods have achieved considerable progress, there still remains a big challenge. Ay *et al*. [10] reported that the methods which only search for one-to-one mappings between molecules could not identify biologically relevant mappings when different organisms perform the same or similar function through a varying number of steps. An example is shown in Fig 1, where both paths transform LL-2,6-diaminopimelate into 2,3,4,5-tetrahydrodipicolinate. The upper path denotes the shortcut used by plants to synthesize L-lysine. Due to the lack of the gene encoding LL-DAP aminotransferase (2.6.1.83) catalyzing reaction R07613, *H*. *sapiens* has to employ a three-step process, as shown with the lower path in Fig 1, to accomplish this transformation. The upper and lower paths should be mapped together in a meaningful alignment when the lysine biosynthesis pathways of human and a plant are aligned. However, due to the different number of reactions in these two paths, traditional methods that are restricted to finding one-to-one mappings fail to uncover the mapping in Fig 1. Motivated by this challenge, researchers develop the other type of alignment methods that allows not only one-to-one mappings but also one-to-many mappings between reactions of two metabolic pathways to tackle this problem. Ay *et al*. [10] proposed for the first time a one-to-many alignment model and an alignment method called SubMAP which searches one-to-many mappings between reactions of two metabolic pathways. SubMAP successfully identifies biologically relevant mappings of alternative subnetworks, and is scalable for metabolic pathways of arbitrary topology. To improve the quality of one-to-many alignments of metabolic pathways, Abaka *et al*. [21] presented a constrained alignment method CAMPways where its goal is to maximize the topological similarity while satisfying some constraints on homological similarity. However, due to the cost in exhaustive search of reaction sets, these two methods do not work well for finding reaction mappings in large-scale metabolic pathways.

In this work, we study the problem of aligning two metabolic pathways, which is briefly described as follows. To align two given metabolic pathways, we want to find a set of one-to-one, one-to-many or many-to-many mappings between reactions, and maximize the sum of the similarity scores of these mappings. The similarity score of such mapping is evaluated as a function of the similarity between the aligned reactions in the mapping (see Section ‘Third Stage’ for details). Recall that one-to-many or many-to-many mappings between reactions are used to identify the mappings of alternative pathways that have similar or the same functions through different sets of reactions [10]. High similarity score indicates that the corresponding alternative pathways perform similar or the same functions with high probability.

Our work is based on the observation that connected relation between reactions can be formalized as binary relation of reactions in the metabolic pathway. Motivated by this observation, we propose an alignment method called MPBR for aligning a pair of metabolic pathways exploiting binary relation of reactions. We formalize connected relation between reactions as binary relation of reactions in metabolic pathway. We exploit for the first time the multiplications of zero-one matrices of binary relation of reactions in finding reaction sets. We show that the multiplications of zero-one matrices of binary relation of reactions can be completed in finite steps. As a consequence, we efficiently obtain such reaction sets in a small number of steps without the need of exhaustive search. Furthermore, distinguishing from measuring the topological similarity of reactions based on the direct neighbors of the reactions [10] or the conserved edges induced by the pairs of reaction mappings in the alignment [21], we measure the topological similarity of nodes (reactions) by comparing the structural similarity of the *k*-neighborhood subgraphs of the nodes, which helps to improve the accuracy of the alignments due to the use of more topological information of the neighbors of the reactions. Our experimental results on the KEGG database show that when compared with other state-of-the-art methods, in most cases, MPBR obtains better topological and biological quality of the alignments than CAMPways and SubMAP, and accurately returns more biologically relevant reaction mappings.

The rest of the paper is organized as follows. Section ‘Method’ presents our method MPBR. Section ‘Results’ shows experimental results. Section ‘Conclusions’ concludes the paper.

## Method

### Preliminaries

**Fig 2:**
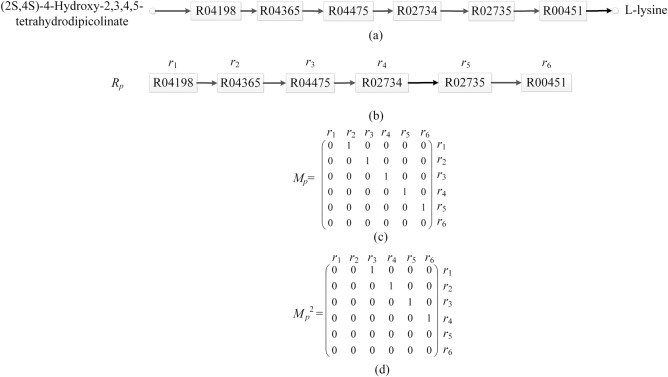
Binary relation of reactions in the metabolic pathway of lysine biosynthesis. The square rectangles represent reactions. The compounds are depicted by small circles. Reactions are represented by their KEGG identifiers. The directed edge from reactions *r*_*i*_ to *r*_*j*_ denotes that at least one output compound of *r*_*i*_ is an input compound of *r*_*j*_. *R*_*p*_ is the binary relation of reactions in the metabolic pathway of lysine biosynthesis. (a) The metabolic pathway of lysine biosynthesis. (b) Directed graph for *R*_*p*_. (c) Zero-one matrix *M*_*p*_ for *R*_*p*_. (d) Zero-one matrix *M*_*p*_^2^.

To start with, we introduce some definitions and notations. A directed graph *G*_*p*_ = (*V*_*p*_,*E*_*p*_) is used to denote metabolic pathway *P*. *V*_*p*_ = {*r*_1_,*r*_2_,…,*r*_*i*_,…,*r*_*k*_} is the node set of *G*_*p*_ and each node *r*_*i*_ represents a reaction in *P*, *i* = 1,2,…,*k*. *E*_*p*_ is the set of directed edges of *G*_*p*_. There is a directed edge (*r*_*i*_, *r*_*j*_)∈*E*_*p*_ from *r*_*i*_ to *r*_*j*_ if and only if at least one output compound of *r*_*i*_ is an input compound of *r*_*j*_, *i* = 1,2,…,*k* and *j* = 1,2,…,*k*. If both *r*_*i*_ and *r*_*j*_ are reversible, there is also a directed edge (*r*_*j*_, *r*_*i*_)∈*E*_*p*_ from *r*_*j*_ to *r*_*i*_. Similarly, a directed graph *G*_*p*_′ = (*V*_*p*_′,*E*_*p*_′) is used to denote metabolic pathway *P*′. Fig 2(A) shows a directed graph for the metabolic pathway of lysine biosynthesis.

For *G*_*p*_ = (*V*_*p*_,*E*_*p*_), let reaction set *RS* = {*r*_1_,*r*_2_,…,*r*_*n*_} of size *n* be a subset of *V*_*p*_ such that the induced subgraph of the reactions in *RS* is linearly connected in the underlying graph, *n* = 1,2,3,…. We represent the set of such reaction sets in *G*_*p*_ as *RS*^*n*^ = {*RS*_1_, *RS*_2_,…, *RS*_*i*_,…, *RS*_*N*_}, where *N* is the number of the reaction sets, *RS*_*i*_ is the *i*th reaction set in *RS*^*n*^ and *RS*_*i*_ has at most *n* reactions, *i* = 1,2,…,*N*. Similarly, for *G*_*p*_′ = (*V*_*p*_′,*E*_*p*_′), let reaction set *RS*′ = {*r*_1_′,*r*_2_′,…,*r*_*m*_′} of size *m* be a subset of *V*_*p*_′ such that the induced subgraph of the reactions in *RS*′ is linearly connected in the underlying graph, *m* = 1,2,3,…. We represent the set of such reaction sets in *G*_*p*_′ as *RS*^*m*^′ = {*RS*_1_′, *RS*_2_′,…, *RS*_*j*_′,…,*RS*_*M*_′}, where *M* is the number of the reaction sets, *RS*_*j*_′ is the *j*th reaction set in *RS*^*m*^′ and *RS*_*j*_′ has at most *m* reactions, *j* = 1,2,…,*M*. Parameters *n* and *m* are determined by the user. Next, we state our problem formally.

Problem Statement: Given two pathways *G*_*p*_ and *G*_*p*_′, we aim to find a set of mappings (*RS*_*i*_, *RS*_*j*_′) between *RS*^*n*^ and *RS*^*m*^′ in the alignment of *G*_*p*_ and *G*_*p*_′ such that the sum of the similarity scores of the mappings is maximized, *i* = 1,2,…,*N* and *j* = 1,2,…,*M*.

In the following, we introduce how to formalize connected relation between reactions as binary relation of reactions in metabolic pathway. A relation between two related elements of two sets is called binary relation [22]. Accordingly, we formalize the binary relation between reactions *A* and *B* as the relation that *A* is connected with *B* in a metabolic pathway. For example, in Fig 2(A), the reactions of the metabolic pathway of lysine biosynthesis are R04198, R04365, R04475, R02734, R02735, and R00451 (reactions are represented by their KEGG identifiers). The relations between two connected reactions in this pathway are represented as (R04198, R04365), (R04365, R04475), (R04475, R02734), (R02734, R02735), and (R02735, R00451). They can be formalized as binary relation {(R04198, R04365), (R04365, R04475), (R04475, R02734), (R02734, R02735), (R02735, R00451)}. As can be seen from Fig 2(B), binary relation of reactions in this pathway can be represented by a directed graph. Also, we can see from Fig 2(C) that this binary relation can be represented by a zero-one matrix *M*_*p*_.

In this work, we represent binary relation of reactions in metabolic pathway by zero-one matrix *M*_*p*_. *M*_*p*_[*i*,*j*] = 1 when reaction *r*_*i*_ is connected to reaction *r*_*j*_, and *M*_*p*_[*i*,*j*] = 0 when *r*_*i*_ is not connected to *r*_*j*_, *i* = 1,2,…,*N* and *j* = 1,2,…,*M*. *M*_*p*_^*n*^ can be computed recursively by *M*_*p*_^1^ = *M*_*p*_ and $M_{p}^{n}=M_{p}^{n-1}\cdot M_{p}$, where $M_{p}^{n-1}\cdot M_{p}$ is a Boolean matrix multiplication, positive integer *n*≥2. Fig 2(D) shows an example of zero-one matrix *M*_*p*_^2^.

In the following section, we present our method MPBR.

### MPBR method

**Fig 3:**
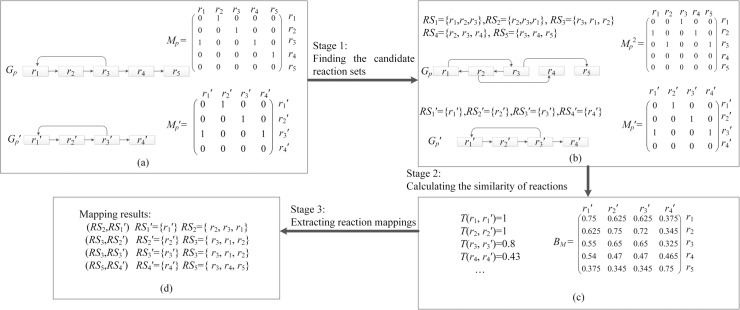
Overview of the MPBR method. MPBR searches 1-to-3 reaction mappings between *G*_*p*_′ and *G*_*p*_. *M*_*p*_ and *M*_*p*_*′* are zero-one matrices for binary relation of reactions in *G*_*p*_ and *G*_*p*_′ respectively. The size of reaction set *RS*_*x*_*′* in *G*_*p*_*′* is *m* = 1, *x* = 1,2,3,4. The size of reaction set *RS*_*y*_ in *G*_*p*_ is *n* = 3, *y* = 1,2,3,4,5. *T*(*r*_1_,*r*_1_*′*), *T*(*r*_2_,*r*_2_*′*), *T*(*r*_3_,*r*_3_′) and *T*(*r*_4_,*r*_4_′) are the topological similarities between the reactions in *G*_*p*_ and *G*_*p*_′ respectively, the values of *B*_*M*_ are the similarities between the reactions in *G*_*p*_ and *G*_*p*_′.

For a pair of metabolic pathways *G*_*p*_ = (*V*_*p*_, *E*_*p*_) and *G*_*p*_′ = (*V*_*p*_′, *E*_*p*_′), the goal of MPBR is to find the reaction mappings between *G*_*p*_ and *G*_*p*_′. Without loss of generality, we assume that |*V*_*p*_|≤|*V*_*p*_′|, reaction sets *RS*⊆*V*_*p*_ and *RS*′⊆*V*_*p*_′. MPBR consists of three main stages (as shown in Fig 3): (1) Find all reaction set *RS* of size *n* for *G*_*p*_, and find all reaction sets *RS*′ of size *m* for *G*_*p*_′ (as detailed in Subsection ‘First Stage’); (2) Construct a similarity matrix *B*_*M*_ by computing the similarity between the reactions in *G*_*p*_ and *G*_*p*_′ (as detailed in Subsection ‘Second Stage’); (3) Find mapping (*RS*, *RS*′) such that the similarity score of mapping (*RS*, *RS*′) is maximized (as detailed in Subsection ‘Third Stage’). A set *RS*_*map*_ of mappings (*RS*, *RS*′) is the result for aligning *G*_*p*_ and *G*_*p*_′. Fig 3 shows an example illustrating the process of aligning a pair of sample pathways.

#### First Stage: Finding the candidate reaction sets

In this subsection, we discuss how to exploit the multiplications of zero-one matrices for binary relation of reactions to create the set *RS*^*n*^ = {*RS*_**1**_, *RS*_**2**_,…, *RS*_*N*_} in *G*_*p*_, and the set *RS*^*m*^′ = {*RS*_**1**_′, *RS*_**2**_′,…, *RS*_*M*_′} in *G*_*p*_′ respectively. For metabolic pathway *G*_*p*_, there is a path from *r*_**1**_ to *r*_*n*_ if there is a sequence of reactions *r*_**1**_,*r*_**2**_,…,*r*_*n*_ with edges (*r*_**1**_, *r*_**2**_), (*r*_**2**_, *r*_**3**_),…, and (*r*_*n*-1_, *r*_*n*_) in *G*_*p*_. Accordingly, we derive theorem 1.

Theorem 1: For reactions *r*_*i*_ and *r*_*j*_, there is a path of length *n* from *r*_*i*_ to *r*_*j*_ in *G*_*p*_ if and only if *M*_*p*_^*n*^[*i*,*j*] = 1, where *n* is a positive integer, *i* = 1,2,…,*n* and *j* = 1,2,…,*n*.

Proof: We will use mathematical induction. There is a path of length one from *r*_*i*_ to *r*_*j*_ in *G*_*p*_ if and only if *M*_*p*_[*i*,*j*] = 1, so the theorem is true when *n* = 1.

Assume that the theorem is true for a positive integer *n*. There is a path of length *n*+1 from *r*_*i*_ to *r*_*j*_ if and only if there is a reaction *r*_*k*_ in *G*_*p*_ such that there is a path of length one from *r*_*i*_ to *r*_*k*_ in *G*_*p*_, so *M*_*p*_[*i*, *k*] = 1, and a path of length *n* from *r*_*k*_ to *r*_*j*_ in *G*_*p*_, that is, *M*_*p*_^*n*^[*k*, *j*] = 1. Consequently, by the induction hypothesis, there is a path of length *n*+1 from *r*_*i*_ to *r*_*j*_ in *G*_*p*_ if and only if there is a reaction *r*_*k*_ with *M*_*p*_[*i*, *k*] = 1 and *M*_*p*_^*n*^[*k*, *j*] = 1. But there is such a reaction if and only if *M*_*p*_^*n*+1^[*i*, *j*] = 1. Therefore, there is a path of length *n*+1 from *r*_*i*_ to *r*_*j*_ in *G*_*p*_ if and only if *M*_*p*_^*n*+1^[*i*, *j*] = 1, *i* = 1,2,…,*n* and *j* = 1,2,…,*n*. γ

Following from theorem 1, we introduce how to find reaction set *RS* of size *n* for *G*_*p*_.

First, we compute *M*_*p*_^*n*-1^. Then we create a reaction pair set *NS* and iteratively extend *NS* with the reaction pair (*r*_*i*_, *r*_*j*_) where *M*_*p*_^*n*-1^[*i*, *j*] = 1. According to theorem 1, for reactions *r*_*i*_ and *r*_*j*_ with *M*_*p*_^*n*-1^[*i*, *j*] = 1, there is a path of length *n*-1 from *r*_*i*_ to *r*_*j*_ in *G*_*p*_ if and only if *M*_*p*_^*n*-1^[*i*, *j*] = 1. That is, there are *n* reactions in this path. Thus, we can construct reaction set *RS* of size *n* containing these *n* reactions. Finally, we search every path of length *n* between two reactions in each reaction pair of *NS* in *G*_*p*_, and create each reaction set *RS* of size *n* containing the reactions in each path to construct the set *RS*^*n*^ = {*RS*_1_, *RS*_2_,…, *RS*_*N*_} in *G*_*p*_. Similarly, we can find each reaction set *RS*′ of size *m* and construct the set *RS*^*m*^′ = {*RS*_1_′, *RS*_2_′,…, *RS*_*M*_′} in *G*_*p*_′.

Fig 3(B) shows an example of reaction sets of size 3. In this example, we first obtain the reaction pairs (*r*_*i*_, *r*_*j*_) with *M*_*p*_^2^[*i*, *j*] = 1, *i* = 1,2,3 and *j* = 1,2,3,4,5. These reaction pairs are (*r*_1_,*r*_3_), (*r*_2_,*r*_1_), (*r*_3_,*r*_2_), (*r*_2_,*r*_4_) and (*r*_3_,*r*_5_). Next we create a reaction pair set *NS* = {(*r*_1_,*r*_3_), (*r*_2_,*r*_1_), (*r*_3_,*r*_2_), (*r*_2_,*r*_4_), (*r*_3_,*r*_5_)} by these reaction pairs. Then, we search the paths of length 2 between two reactions in each reaction pair of *NS*. These paths are Path 1(*r*_1_→*r*_2_→*r*_3_), Path 2 (*r*_2_→*r*_3_→*r*_1_), Path 3 (*r*_3_→*r*_1_→*r*_2_), Path 4 (*r*_2_→*r*_3_→*r*_4_) and Path 5 (*r*_3_→*r*_4_→*r*_5_) respectively. Finally we create reaction sets *RS*_1_ = {*r*_1_,*r*_2_,*r*_3_}, *RS*_2_ = {*r*_2_,*r*_3_,*r*_1_}, *RS*_3_ = {*r*_3_, *r*_1_, *r*_2_}, *RS*_4_ = {*r*_2_, *r*_3_, *r*_4_} and *RS*_5_ = {*r*_3_, *r*_4_, *r*_5_} with the reactions in Path 1, Path 2, Path 3, Path 4, and Path 5 respectively.

Based on theorem 1 and the above searching procedure of reaction sets in *G*_*p*_, we drive the following property.

Property 1: Reaction set *RS* of size *n*+1 in *G*_*p*_ can be found through performing *M*_*p*_^*n*^.

Property 1 illustrates the relationship between the search of reaction sets and the multiplications of zero-one matrices for binary relation of reactions in *G*_*p*_.

For *M*_*p*_^*n*^, we have the following theorem.

Theorem 2: If there exist positive integers *t* and *s* with *t*>*s* such that *M*_*p*_^*t*^ = *M*_*p*_^*s*^, then for any positive integer *n*, it holds that *M*_*p*_^*n*^∈*S* = {*M*_*p*_, *M*_*p*_^2^, …, *M*_*p*_^*t-*1^}.

Proof:

For any *n*, when *n*≤*t*-1, we have *M*_*p*_^*n*^∈*S*. We want to prove *M*_*p*_^*n*^∈*S* when *n*>*t*-1. When *n*>*t*-1, it holds *n*>*s*. For *n*, *s* and *t*, there exist positive integers *q* and *r* such that *n*-*s* = (*t*-*s*)*q*+*r* (0<*r*≤*t*-*s*-1,*n*>*s*). Therefore, *n* = *s*+(*t*-*s*)*q*+*r* and *M*_*p*_^*n*^ = *M*_*p*_^*s*+(*t*-*s*)*q*+*r*^. Since 0<*r*≤*t*-*s*-1, so *s*+*r*≤*t*-1 and *M*_*p*_^*s*+*r*^∈*S*. Since *M*_*p*_^*n*^ = *M*_*p*_^*s*+(*t*-*s*)*q*+*r*^, if we can prove *M*_*p*_^*s*+(*t*-*s*)*q*+*r*^ = *M*_*p*_^*s*+*r*^, then it follows *M*_*p*_^*n*^ = *M*_*p*_^*s*+*r*^∈*S*. In the following, we will use mathematical induction to prove *M*_*p*_^*s*+(*t*-*s*)*q*+*r*^ = *M*_*p*_^*s*+*r*^.

When *q* = 1, it yields *M*_*p*_^*s*+(*t-s*)*q*+*r*^ = *M*_*p*_^*s*+(*t*-*s*)+*r*^ = *M*_*p*_^*t*+*r*^ = *M*_*p*_^*s*+*r*^. Assume that for any *q*≤*k*, *M*_*p*_^*s*+(*t*-*s*)*q*+*r*^ = *M*_*p*_^*s*+*r*^ holds. When *q* = *k*+1, we have *M*_*p*_^*s*+(*t*-*s*)(*k*+1)+*r*^ = *M*_*p*_^*s*+(*t*-*s*)*k*+*r*+(*t*-*s*)^ = *M*_*p*_^*s+*(*t-s*)*k+r*^*M*_*p*_^(*t*-*s*)^ = *M*_*p*_^*s*+*r*^*M*_*p*_^*t*-*s*^ = *M*_*p*_^*s*+*r*+*t*-*s*^ = *M*_*p*_^*t*+*r*^ = *M*_*p*_^*s*+*r*^. Hence, *M*_*p*_^*s*+(*t*-*s*)*q*+*r*^ = *M*_*p*_^*s*+*r*^ also holds for *q* = *k*+1. By induction, we have proven that *M*_*p*_^*s*+(*t*-*s*)*q*+*r*^ = *M*_*p*_^*s*+*r*^ for all *q*>0. Consequently, since *M*_*p*_^*n*^ = *M*_*p*_^*s*+(*t*-*s*)*q*+*r*^, *M*_*p*_^*s*+(*t*-*s*)*q*+*r*^ = *M*_*p*_^*s*+*r*^ and *M*_*p*_^*s*+*r*^∈*S*, hence *M*_*p*_^*n*^ = *M*_*p*_^*s*+*r*^∈*S*.ϒ

The discussion regarding the search of reaction sets in *G*_*p*_ and theorem 2 leads to the following corollary.

Corollary 1: If there exist positive integers *s* and *t* with *s*<*t* such that *M*_*p*_^*s*^ = *M*_*p*_^*t*^, then the reaction set *RS* of size *n*≥*t* in *G*_*p*_ can be found through performing at most *M*_*p*_^*t*-1^.

Proof:

Let *S* = {*M*_*p*_, *M*_*p*_^2^,…,*M*_*p*_^*t*-1^}. When there exist positive integers *s* and *t* with *s*<*t* such that *M*_*p*_^*s*^ = *M*_*p*_^*t*^, according to theorem 2, for any positive integer *n*, it holds that *M*_*p*_^*n*^∈*S* = {*M*_*p*_, *M*_*p*_^2^,…,*M*_*p*_^*t*-1^}. In other words, when *n*-1≥*t*-1, there is a positive integer *v*≤*t*-1 such that *M*_*p*_^*v*^ = *M*_*p*_^*n*-1^∈*S*. According to property 1, we need to perform *M*_*p*_^*n*-1^ to find reaction set *RS* of size *n* in *G*_*p*_. Because *M*_*p*_^*v*^ = *M*_*p*_^*n*-1^ when *n*-1≥*t*-1, so we only need to perform *M*_*p*_^*v*^ to find the reaction set *RS* of size *n* in *G*_*p*_ instead of performing *M*_*p*_^*n*-1^, where *v*≤*t*-1. ϒ

Property 1 implies that we can find reaction set *RS* of size *n* through performing *M*_*p*_^*n*-1^. Furthermore, corollary 1 implies that, in order to find reaction set *RS* of size *n*, when *n*≥*t*, we only need to try at most *M*_*p*_^*t*-1^. Therefore, we can find reaction set *RS* of size greater than *t* in metabolic pathway through performing at most *M*_*p*_^*t*-1^.

In the first stage, MPBR first computes *M*_*p*_^*n*-1^. Secondly, MPBR creates a reaction pair set *NS* and iteratively extends *NS* with the reaction pair (*r*_*i*_, *r*_*j*_) where *M*_*p*_^*n*-1^[*i*,*j*] = 1. Finally, through finding the paths of length *n*-1 between two reactions in each reaction pair of *NS* in *G*_*p*_, MPBR obtains the set *RS*^*n*^ = {*RS*_1_, *RS*_2_,…, *RS*_*N*_} in *G*_*p*_ and the set *RS*^*m*^′ = {*RS*_1_′, *RS*_2_′,…, *RS*_*M*_′} in *G*_*p*_′. Thus, in this way, we avoid the exhaustive search of reaction sets, and produce candidate reaction sets in finite steps.

#### Second Stage: Calculating the similarity of reactions

The second stage aims to compute the similarity values between any two reactions in *G*_*p*_ and *G*_*p*_*′*, which combines topological and homological similarities of reactions, and construct a |*V*_*p*_|×|*V*_*p*_*′*| similarity matrix *B*_*M*_, where *B*_*M*_ [*u*,*v*] is the similarity value between nodes (reactions) *u* and *v*, *u*∈*V*_*p*_, *v*∈*V*_*p*_*′*.

We first introduce how to compute topological similarity of nodes (reactions) in metabolic pathways. Our computation of topological similarity of nodes is based on the following observation. If node *u* is mapped to node *v*, then their neighbors in the corresponding graphs should also be similar. From this observation, we measure topological similarity of nodes by comparing the structural similarity of the *k*-neighborhood subgraphs of nodes. Next, we discuss how to compare the *k*-neighborhood subgraphs of nodes to compute topological similarity of nodes. *N*_*k*_(*u*) is defined as the *k*-neighborhood node set of *u* in *G*_*p*_ and *N*_*k*_(*u*) is a subset of *V*_*p*_, *u*∉*N*_*k*_(*u*), where *k* is an integer and *k*≥0. The shortest distance between *u* and *x*∈*N*_*k*_(*u*) is defined as the number of edges of the shortest path between *u* and *x*, which is not exceeding *k*. Similarly, *N*_*k*_(*v*) is defined as the *k*-neighborhood node set of *v* in *G*_*p*_′.

For *u*∈*V*_*p*_ and *v*∈*V*_*p*_*′*, the *k*-neighborhood subgraph of *u* in *G*_*p*_ is denoted by $G_{u}^{k}$. *E*_*k*_(*u*) is defined as the edge set of $G_{u}^{k}$. $G_{u}^{k}$ is the induced subgraph over *N*_*k*_(*u*)∪{*u*} in *G*_*p*_. The *k*-neighborhood subgraph of *v* in *G*_*p*_′ is denoted by Gvk'. Gvk' is the induced subgraph over *N*_*k*_(*v*)∪{*v*} in *G*_*p*_′. Let *d*(*u*) be the degree of *u* in *G*_*p*_ and *d*(*v*) be the degree of *v* in *G*_*p*_′. Suppose that the degree sequence of the nodes in *N*_*k*_(*u*) is *d*(*x*_1_), *d*(*x*_2_),…,*d*(*x*_*i*_),…,and $d(x_{\left|N_{k}(u)\right|})$ sorted in a non-increasing order, and the degree sequence of the nodes in *N*_*k*_(*v*) is *d*(*y*_1_), *d*(*y*_2_),…,*d*(*y*_*j*_),…,and $d(x_{\left|N_{k}(v)\right|})$ sorted in non-increasing order. We compare the *k*-neighborhood subgraph of *u* with the *k*-neighborhood subgraph of *v* to compute the topological similarity *T*(*u*,*v*) between *u* and *v*:

T(u,v)=min{|V(Guk)|,|V(Gvk')|}+12∑k=0Kmmin{∑1≤i≤|Nk(u)|∧xi∈Nk(u)d(xi),∑1≤i≤|Nk(v)|∧yi∈Nk(v)d(yi)}max{|V(Guk)|,|V(Gvk')|}+max{|E(Guk)|,|E(Gvk')|} (1)

where *K*_*m*_ is the maximal value of *k* and is determined by the user, $\sum_{1\leq i\leq |N_{k}(u)|\wedge x_{i}\in N_{k}(u)}d(x_{i})$ and $\sum_{1\leq i\leq |N_{k}(v)|\wedge y_{i}\in N_{k}(v)}d(y_{i})$ are the sum of degrees of nodes in *N*_*k*_(*u*) and *N*_*k*_(*v*) respectively. The impact of edges on the topological similarity *T*(*u*,*v*) is evaluated by $\sum_{1\leq i\leq |N_{k}(u)|\wedge x_{i}\in N_{k}(u)}d(x_{i})$ and $\sum_{1\leq i\leq |N_{k}(v)|\wedge y_{i}\in N_{k}(v)}d(y_{i})$. When *k* = 0, *N*_*k*_(*u*) = *u* and *N*_*k*_(*v*) = *v*.

In the following, we discuss how to compute homological similarity between reactions. Since reactions consist of the input and output compounds and enzymes, we measure the homological similarity between reactions by the similarities of these components. Thus the homological similarity *Bsim*(*u*,*v*) between *u* and *v* is computed by the following equation:

$$Bsim\left(u,v\right)=\alpha \times Esim\left(u_{e},v_{e}\right)+\beta \times Csim\left(u_{i},v_{i}\right)+\gamma \times Csim\left(u_{o},v_{o}\right)$$ (2)

where *u*_*e*_ is the enzyme catalyzing reaction *u*, *v*_*e*_ is the enzyme catalyzing reaction *v*, *Esim*(*u*_*e*_,*v*_*e*_) is the similarity score between enzyme *u*_*e*_ and enzyme *v*_*e*_. We employ the enzyme similarity score defined in [16] to calculate *Esim*(*u*_*e*_,*v*_*e*_). More specifically, the EC identifier of an enzyme consists of four digits that categorize the type of the catalyzed chemical reaction. *Esim*(*u*_*e*_,*v*_*e*_) is 1 if all the four digits of the EC identifier of two enzymes are identical, *Esim*(*u*_*e*_,*v*_*e*_) is 0.75 if the first three digits are identical, *Esim*(*u*_*e*_,*v*_*e*_) is 0.5 if the first two digits are identical, *Esim*(*u*_*e*_,*v*_*e*_) is 0.25 if the first digit is identical, and *Esim*(*u*_*e*_,*v*_*e*_) is 0 if the first digit is different [16]. For example, for enzymes 2.3.1.117 and 2.6.1.17, *Esim*(2.6.1.18, 2.6.1.12) = 0.75. The input compounds of *u* and *v* are *u*_*i*_ and *v*_*i*_ respectively, and the output compounds of *u* and *v* are *u*_*o*_ and *v*_*o*_ respectively. *Csim*(*u*_*i*_,*v*_*i*_) is the average similarity score of compounds *u*_*i*_ and *v*_*i*_, and *Csim*(*u*_*o*_,*v*_*o*_) is the average similarity score of compounds *u*_*o*_ and *v*_*o*_. For example, if *C*_1_ and *C*_2_ are the input compounds of *u*, and *C*_3_ and *C*_4_ are the input compounds of *v*, then *Csim*(*u*_*i*_,*v*_*i*_) = {*sim*(*C*_1_,*C*_3_)+*sim*(*C*_1_, *C*_4_)+*sim*(*C*_2_, *C*_3_)+*sim*(*C*_2_, *C*_4_)}/4, where *sim*(*A*, *B*) is the similarity score of compounds *A* and *B*. Similarly, we can compute *Csim*(*u*_*o*_,*v*_*o*_). The similarity scores of compounds are calculated by the SIMCOMP package [23]. For example, the similarity score of compounds acetoacetyl-CoA and (S)-3-Hydroxy-3-methylglutaryl-CoA is 0.723077. Parameters *α*, *β* and *γ* control the balance between the weights of *Esim*(*u*_*e*_,*v*_*e*_), *Csim*(*u*_*i*_,*v*_*i*_) and *Csim*(*u*_*o*_,*v*_*o*_) with the constraint *α*+*β*+*γ* = 1. For the choice of weight parameters *α*, *β* and *γ*, we use *α* = 0.4, *β* = 0.3 and *γ* = 0.3.

Both homological and topological similarities of reactions provide significant information for the alignment of metabolic pathways. We are now ready to define our similarity *S*(*u*,*v*) between *u* and *v*, which is computed by the following equation:

S(u,v)=σ×T(u,v)+(1-σ)×Bsim(u,v) (3)

where *σ* is a balancing parameter between the weight of *T*(*u*,*v*) and the weight of *Bsim*(*u*,*v*), 0≤*σ*≤1.

In the second stage, we use Eq (3) to calculate the similarity values between any two reactions in two pathways, and construct a similarity matrix *B*_*M*_ for the reactions using these similarity values. For example, when *k* = 2, *σ* = 0.5, for simplicity, we assume that the homological similarities between any two reactions in sample pathways *G*_*p*_ and *G*_*p*_′ are 0.5, a similarity matrix *B*_*M*_ for the reactions in *G*_*p*_ and *G*_*p*_′ is shown in Fig 3(C).

#### Third Stage: Extracting reaction mappings

Once we obtain the set *RS*^*n*^ = {*RS*_**1**_, *RS*_**2**_,…, *RS*_*i*_,…, *RS*_*N*_} in *G*_*p*_, the set *RS*^*m*^′ = {*RS*_**1**_′, *RS*_**2**_′,…, *RS*_*j*_′,…,*RS*_*M*_′} in *G*_*p*_′, and the similarity matrix *B*_*M*_ for the reactions, we try to extract mappings (*RS*_*i*_, *RS*_*j*_′) that constitute our alignment. In the third stage, for each reaction set in *RS*^*n*^ and *RS*^*m*^′, we first perform greedy search to find a mapping (*RS*_*i*_, *RS*_*j*_′) such that the similarity score of mapping (*RS*_*i*_, *RS*_*j*_′) is maximized, and then add mapping (*RS*_*i*_,*RS*_*j*_′) to the set *RS*_*map*_ of mappings. To compute the similarity score of mapping (*RS*_*i*_, *RS*_*j*_′), we obtain the similarity value *S*_**1**_ between the start reactions in *RS*_*i*_ and *RS*_*j*_′, and the similarity value *S*_**2**_ between the end reactions in *RS*_*i*_ and *RS*_*j*_′ from similarity matrix *B*_*M*_ obtained in the second stage. The average value of *S*_**1**_ and *S*_**2**_ is the similarity score of mapping (*RS*_*i*_, *RS*_*j*_′). For example, as can be seen from Fig 3(C), in the similarity matrix *B*_*M*_, the similarity value between the start reactions in *RS*_**2**_ and *RS*_**1**_′ is 0.625, and the similarity value between the end reactions in *RS*_**2**_ and *RS*_**1**_′ is 0.75. Then the similarity score of mapping (*RS*_**2**_, *RS*_**1**_′) is 0.6875.

The greedy search for mappings (*RS*_*i*_, *RS*_*j*_′) is repeated until all reaction sets in *RS*^*n*^ are aligned with the reaction sets in *RS*^*m*^′. At this time, the set *RS*_*map*_ of mappings (*RS*_*i*_, *RS*_*j*_′) is the result of aligning *G*_*p*_ and *G*_*p*_′. Fig 3(D) shows an example of the mapping results found in the alignment of a pair of sample pathways.

In summary, we first utilize the multiplications of zero-one matrices for binary relation of reactions to find reaction set *RS* of size *n* for *G*_*p*_ and reaction set *RS*′ of size *m* for *G*_*p*_′. Second, in order to improve the topological and biological accuracy of the alignments for metabolic pathways, we propose a measure of topological similarity of nodes (reactions), which compares the structural similarity of the *k*-neighborhood subgraphs of the nodes. Then, we measure the similarity between reactions by combining topological and homological similarities of reactions and build a similarity matrix *B*_*M*_ between all reactions in two pathways. Finally, we employ a greedy search to find a set of reaction mappings (*RS*, *RS*′) where the sum of the similarity scores of each mapping is maximized.

## Results

MPBR is implemented in Java, the data and program are available at http://210.36.16.170:8080/MPBR/MPBR.zip. Currently, CAMPways and SubMAP are the two available alignment softwares that allow one-to-many reaction mappings in the alignment of metabolic pathways. We downloaded CAMPways and SubMAP from http://code.google.com/p/campways/ and http://bioinformatics.cise.ufl.edu/SubMAP.html respectively. In this section, we experimentally compared the performance of MPBR with that of CAMPways and SubMAP, and discussed three sample alignments.

The KEGG database [1] provides 11 metabolism categories: 1.1 carbohydrate metabolism, 1.2 energy metabolism, 1.3 lipid metabolism, 1.4 nucleotide metabolism, 1.5 amino acid metabolism, 1.6 metabolism of other amino acids, 1.7 glycan biosynthesis and metabolism, 1.8 metabolism of cofactors and vitamins, 1.9 metabolism of terpenoids and polyketides, 1.10 biosynthesis of other secondary metabolites, and 1.11 xenobiotics biodegradation and metabolism.

From the metabolic pathways of the KEGG database retrieved and reformatted by Ay *et al*. [10], Abaka *et al*.[21] provided a dataset of 11 metabolic pathways to evaluate alignment quality. Each pathway in this dataset corresponds to one of the above metabolisms. Following the state-of-the-art method CAMPways [21], we also evaluate alignment quality using this dataset. The experimental evaluations are divided into the pathway alignments between species within the same domain and the pathway alignments between species from different domains. Similar to CAMPways, *Homo sapiens* (*hsa*) and *Mus musculus* (*mmu*) are selected as two representative species from the eukaryota domain, while *Escherichia coli* (*eco*) and *Agrobacterium tumefaciens* (*atc*) are selected as two representative species from the bacteria domain.

By using the method proposed by Abaka *et al*. [21], we merge all pathways of the above metabolisms into a large pathway for each of these four species. Thus, we totally obtain four large merged pathways, namely *hsa-*1.12 with 1520 nodes, *mmu-*1.12 with 1466 nodes, *eco-*1.12 with 1104 nodes and *atc-*1.12 with 1127 nodes. We also use these four large merged pathways to evaluate the performance of the alignment methods. Moreover, we use eight real metabolic pathways *eco*00230, *eco*00240, *hsa*00230, *hsa*00240, *atc*00230, *atc*00240, *mmu*00230 and *mmu*00240 from these four species as test pathways. These eight metabolic pathways are obtained from the literature [10] and they are represented by *eco-*1.13, *eco-*1.14, *hsa-*1.13, *hsa-*1.14, *atc-*1.13, *atc-*1.14, *mmu-*1.13 and *mmu-*1.14 respectively in this paper. S1 Table presents the number of nodes and the number of edges of the pathways used in the experiments.

The experimental comparisons are conducted based on six criteria. Next, we introduce these criteria in detail [10, 21, 24–26].

1 *Edge Correctness* (*EC*) is the percentage of the edges of the first pathway that are aligned to the edges of the second pathway. The more similar topology of the two pathways, the higher value of the *EC* [24]. *EC* is calculated by the following equation [24–26]:
  $$EC=\frac{\left|\{(u,v)\in E_{1}:(g(u),g(v))\in E_{2}\}\right|}{\left|E\right|}$$
  where *u* and *v* are the nodes in the first pathway, (*u*,*v*) is an edge in the first pathway, *E* is the edge set of the first pathway, *E*_1_ is the matched edge set of the first and second pathways, *g*(*u*) and *g*(*v*) are the mapping nodes of *u* and *v* in the second pathway respectively, and *E*_2_ is the edge set of the second pathway.
2 *Node Correctness* (*NC*) is the percentage of nodes of the first pathway that are aligned to the correct nodes of the second pathway. *NC* is calculated by the following equation [24]:
  $$NC=\frac{\left|\{u\in V_{1}:f(u)=g(u)\}\right|}{\left|V_{1}\right|}$$
  where *u* is a node in the first pathway, *V*_1_ is the node set of the first pathway, *f* is the correct node mapping, and *g* is the alignment mapping. For the correct node mapping *f*, we use measurement FGC (functional group conversion category), which was previously used to define the correct mapping between pathways in [21], to judge whether the node mapping is correct. Specifically, FGC category is a part of the RCLASS database [27] of KEGG. The reactions in the KEGG database are classified into hierarchically organized functional group categories [21]. There are eight FGC categorizations of the KEGG hierarchy, and each FGC categorization is divided into five levels. Abaka *et al*. pointed out that an inter-species alignment of a pair of pathways is considered biologically validated if the alignment maps reaction subsets classified under the same FGC category [21]. The biological relevance of reaction mappings is closely related to the FGC hierarchy of reactions in the mappings. More specifically, a reaction mapping is biologically more significant if it includes more reactions with higher FGC hierarchy under the same FGC category. In the experimental results of the main text, for a fixed level 5 of the hierarchy, a node mapping is called correct if there exists at least one category at the 5^th^ level of the FGC hierarchy that includes all the reactions in the mapping [21].
3 *The Number of Edges of Largest Common Connected Subgraph* (*ELCCS*) is the number of the edges of the largest connected subgraph of the first pathway that is isomorphic to a subgraph of the second pathway [24]. *ELCCS* is used to evaluate the topological accuracy and biological relevance of the alignments. The larger and denser connected subgraphs are biologically more valuable [24].
4 *C-1tomany* is the number of correct one-to-many reaction mappings between the first pathway and the second one. To describe this measurement, we introduce some notations first. Let *X*, *X′* denote two species and *G*_*X*_, *G*_*X*_′ represent their metabolic pathways corresponding to some metabolism 1.*m*, listed earlier in the text. Let (*RS*, *RS*′) be a mapping from an alignment of *G*_*X*_ and *G*_*X*_′ where *RS* = {*r*_1_}, *RS*′ = {*r*_1_′, *r*_2_′,…,*r*_*x*_′}, and *P*_1_,…,*P*_*i*_,…,*P*_*x*_ be the pathways that include reaction *r*_1_ and are associated with metabolism 1.*m* in the species *X* [21].

Following the literatures [10] and [21], we measure the correctness of the one-to-many reaction mappings based on two aspects. On the one hand, as Ay *et al*. [10] reported that if both alternative pathways can complete the transformations between two given compounds through different reaction sets, then these two pathways are considered to be functionally similar. A correct one-to-many reaction mapping between different pathways should be able to identify the mapping of such alternative pathways [10]. On the other hand, Abaka *et al*. [21] pointed out that an alignment mapping reactions that belong back to the same original KEGG pathway is considered to be of high quality. Combining these two aspects, a one-to-many reaction mapping (*RS*, *RS*′) is called correct if it satisfies the following two conditions: (1) The start reactions in *RS* and *RS*′ share at least one input compound and the end reactions in *RS* and *RS*′ share at least one output compound. (2) Every reaction in *RS*′ is included in at least one of the pathways *P*_1_′,…,*P*_*i*_′,…, *P*_*x*_′ where each *P*_*i*_′ is a pathway in metabolism 1.*m* of species *X′* and corresponds to *P*_*i*_ of *X* [21].

5 *CR-1tomany* is the ratio of the number of correct one-to-many reaction mappings to the total number of mappings produced by the alignment [21]. *CR-1tomany* can be used to investigate the percentage of the correct one-to-many reaction mappings in the alignment. Higher values for *CR-1tomany* indicate that the alignments for one-to-many reaction mapping are of high quality [21].
6 *C-manytomany* is the number of correct many-to-many reaction mappings between the first pathway and the second one. By reference to *C-1tomany*, let (*RS*, *RS*′) be a many-to-many mapping from an alignment of *G*_*X*_ and *G*_*X*_′ where *RS* = {*r*_1_, *r*_2_,…,*r*_*x*_}, *RS*′ = {*r*_1_′, *r*_2_′,…,*r*_*y*_′}, and *P*_1_,…,*P*_*i*_,…,*P*_*x*_ be the pathways that include a reaction in *RS* and are associated with metabolism 1.*m* in the species *X*. Similar to *C-1tomany*, a many-to-many reaction mapping (*RS*, *RS*′) is called correct if it satisfies the following two conditions: (1) The start reactions in *RS* and *RS*′ share at least one input compound and the end reactions in *RS* and *RS*′ share at least one output compound. (2) Every reaction in *RS*′ is included in at least one of the pathways *P*_1_′,…,*P*_*i*_′,…,*P*_*x*_′ where each *P*_*i*_′ is a pathway in metabolism 1.*m* of species *X′* and corresponds to *P*_*i*_ of *X*.

MPBR, CAMPways and SubMAP provide the options of one-to-one alignment and one-to-many alignment. We can perform one-to-one alignment of two pathways to find one-to-one reaction mappings between these two pathways. Similarly, we can perform one-to-many alignment of two pathways to find one-to-many reaction mappings between these two pathways. In the experiments, the one-to-many reaction mappings include 1-to-2 and 1-to-3 reaction mappings.

In this paper, we use *EC*, *NC* and *ELCCS* to measure the quality of one-to-one alignment, and use *C-1tomany* and *CR-1tomany* to measure the quality of one-to-many alignment. In addition, we use *C-manytomany* to evaluate the capability of MPBR for searching many-to-many reaction mappings.

In the experiments, MPBR was run using *k* = 3 and *σ* = 0.6, and CAMPways and SubMAP were run using their default parameter settings. MPBR, CAMPways and SubMAP were run on the Sugon 5000A computer system of cluster architecture at Guangxi University, using a single computing node with a quad-core Intel(R) Xeon(R) CPU E5620 @ 2.40GHz and 40GB RAM. The operating system is Linux.

The following five subsections will provide our experimental evaluations on the qualities of the alignment results computed by MPBR, CAMPways and SubMAP respectively. Subsections ‘Same-domain One-to-one Alignments’ and ‘Same-domain One-to-many Alignments’ focus on one-to-one alignment and one-to-many alignment between the species within the same domain respectively. Subsections ‘Across-domains One-to-one Alignments’ and ‘Across-domains One-to-many Alignments’ focus on one-to-one alignment and one-to-many alignment between the species from different domains respectively. Subsection ‘Running time and memory requirements’ discusses the performance of each method in terms of the running time and memory requirements. Subsection ‘Many-to-many Alignments’ discusses the experimental results of many-to-many alignments of the pathways. Subsection ‘Case study’ introduces three sample alignments to show how MPBR, CAMPways and SubMAP can be used to analyze metabolic pathways.

The values of *NC* of the alignment results of MPBR, CAMPways and SubMAP for the fifth level of the FGC hierarchy are shown in Tables 1–15, whereas the values of *NC* for the first four levels of the FGC hierarchy are shown in S2–S5 Tables.

**Table 1:** *EC* and *NC* of one-to-one alignment results for *eco*-*atc*.

*Pathways*	*EC*			*NC*		
	MPBR	CAMPways	SubMAP	MPBR	CAMPways	SubMAP
1.1	**0.53**	0.26	0.26	**0.53**	**0.53**	**0.53**
1.2	**0.67**	**0.67**	0.00	**0.69**	**0.69**	0.00
1.3	**0.81**	0.58	0.00	**0.71**	0.64	0.00
1.4	**0.75**	0.51	0.00	**0.79**	0.77	0.00
1.5	**0.96**	0.91	0.91	**0.77**	0.76	0.76
1.6	**0.83**	0.67	0.00	**0.80**	0.76	0.00
1.7	**0.77**	0.64	0.65	**0.71**	0.69	0.69
1.8	**0.59**	0.58	0.00	**0.66**	0.61	0.00
1.9	**0.80**	0.70	0.00	**0.80**	0.66	0.00
1.1	**0.88**	0.87	0.00	**0.84**	0.80	0.00
1.11	**0.67**	**0.67**	**0.67**	**0.59**	**0.59**	**0.59**
1.12	**0.85**	0.67	0.65	**0.78**	0.73	0.74
1.13	**0.93**	0.76	0.76	**0.87**	0.84	0.84
1.14	**0.66**	0.47	0.37	**0.71**	0.65	0.58

The best performer for the relative item is marked in bold.

**Table 2:** *EC* and *NC* of one-to-one alignment results for *hsa*-*mmu*.

*Pathways*	*EC*			*NC*		
	MPBR	CAMPways	SubMAP	MPBR	CAMPways	SubMAP
1.1	**1.00**	**1.00**	**1.00**	**0.93**	**0.93**	**0.93**
1.2	**1.00**	**1.00**	0.00	**0.94**	**0.94**	0.00
1.3	**0.99**	0.97	0.97	**0.99**	0.97	0.98
1.4	**1.00**	**1.00**	**1.00**	**1.00**	**1.00**	**1.00**
1.5	**0.94**	0.92	0.92	**0.95**	0.91	0.93
1.6	**0.96**	**0.96**	0.51	**0.99**	**0.99**	0.61
1.7	**0.93**	0.92	0.92	**0.95**	0.93	0.93
1.8	**0.92**	0.91	0.91	**0.93**	**0.93**	**0.93**
1.9	**1.00**	**1.00**	**1.00**	**1.00**	0.68	0.89
1.1	**0.99**	0.97	0.97	**0.98**	0.97	**0.98**
1.11	**1.00**	**1.00**	**1.00**	**0.82**	**0.82**	**0.82**
1.12	**0.97**	0.94	0.94	**0.96**	0.92	0.94
1.13	**0.99**	**0.99**	0.00	**0.99**	**0.99**	0.00
1.14	**1.00**	0.91	0.88	**1.00**	0.99	0.97

The best performer for the relative item is marked in bold.

**Table 3:** *ELCCS* of one-to-one alignment results for *eco-atc* and *hsa-mmu*.

*Pathways*	*eco-atc*			*hsa-mmu*		
	MPBR	CAMPways	SubMAP	MPBR	CAMPways	SubMAP
1.1	**9**	5	4	**14**	**14**	**14**
1.2	**3**	**3**	0	**5**	**5**	0
1.3	**986**	751	0	**763**	750	750
1.4	**99**	73	0	**23**	**23**	**23**
1.5	**191**	**191**	**191**	**459**	289	289
1.6	**534**	364	0	**526**	508	508
1.7	**196**	143	141	**374**	175	176
1.8	**48**	37	0	**57**	50	54
1.9	**12**	**12**	0	**3**	**3**	**3**
1.10	**155**	149	0	**215**	**215**	**215**
1.11	**4**	**4**	**4**	**5**	**5**	**5**
1.12	**3039**	2941	2944	**2878**	2752	2753
1.13	**316**	247	256	**294**	292	0
1.14	**191**	92	95	**224**	204	199

The best performer for the relative item is marked in bold.

**Table 4:** *C-1tomany* and *CR-1tomany* of one-to-many alignment results for *eco*-*atc*.

*Pathways*	*C-1tomany*			*CR-1tomany*		
	MPBR	CAMPways	SubMAP	MPBR	CAMPways	SubMAP
1.1	**0**	**0**	**0**	**0.00**	**0.00**	**0.00**
1.2	**0**	**0**	**0**	**0.00**	**0.00**	**0.00**
1.3	**6274**	34	*	0.34	**0.45**	*
1.4	**117**	1	0	**0.83**	0.07	0.00
1.5	**447**	30	5	0.44	**0.68**	0.50
1.6	**2671**	21	*	**0.89**	0.66	*
1.7	20	**24**	14	0.07	**0.48**	0.42
1.8	**37**	4	1	**0.86**	0.33	0.20
1.9	0	**3**	**3**	0.00	0.25	**0.75**
1.10	**2364**	17	2	**0.66**	0.28	0.11
1.11	**2**	1	0	**1.00**	0.17	0.00
1.12	**12959**	112	*	0.23	**0.40**	*
1.13	**2213**	16	6	**1.00**	0.62	0.35
1.14	**442**	9	6	**1.00**	0.82	0.50

The best performer for the relative item is marked in bold. The asterisk “*” denotes that the program is unable to generate a result under our current computing environment.

**Table 5:** *C-1tomany* and *CR-1tomany* of one-to-many alignment results for *hsa*-*mmu*.

*Pathways*	*C-1tomany*			*CR-1tomany*		
	MPBR	CAMPways	SubMAP	MPBR	CAMPways	SubMAP
1.1	**4**	0	1	**1.00**	0.00	0.13
1.2	**0**	**0**	**0**	**0.00**	**0.00**	**0.00**
1.3	**6117**	43	*	0.57	**0.60**	*
1.4	**16**	2	1	0.31	0.29	**0.50**
1.5	**2195**	33	*	**0.55**	0.35	*
1.6	**421**	19	*	0.13	**0.66**	*
1.7	**1103**	24	6	**0.88**	0.35	0.29
1.8	**85**	4	1	**0.77**	0.24	0.20
1.9	**0**	**0**	**0**	**0.00**	**0.00**	**0.00**
1.10	**5234**	17	*	**0.72**	0.33	*
1.11	**5**	0	0	**1.00**	0.00	0.00
1.12	**18877**	117	*	**0.46**	0.34	*
1.13	**151**	11	*	0.16	**0.61**	*
1.14	**2846**	9	3	**1.00**	0.75	0.38

The best performer for the relative item is marked in bold. The asterisk “*” denotes that the program is unable to generate a result under our current computing environment.

**Table 6:** *EC* and *NC* of one-to-one alignment results for *hsa*-*eco*.

*Pathways*	*EC*			*NC*		
	MPBR	CAMPways	SubMAP	MPBR	CAMPways	SubMAP
1.1	**0.30**	0.16	*	0.45	**0.46**	*
1.2	0.14	**0.29**	0.00	**0.35**	0.18	0.29
1.3	**0.76**	0.48	*	**0.57**	0.54	*
1.4	**0.55**	0.26	*	**0.39**	0.34	*
1.5	**0.47**	0.31	*	**0.28**	0.26	*
1.6	**0.89**	0.72	0.00	**0.81**	0.78	0.00
1.7	**0.42**	0.27	0.25	**0.40**	0.35	0.37
1.8	**0.65**	0.53	*	0.57	**0.58**	*
1.9	**0.00**	**0.00**	**0.00**	**0.08**	0.06	**0.08**
1.1	**0.56**	0.53	0.00	**0.56**	0.55	0.00
1.11	**0.42**	0.33	0.00	**0.36**	**0.36**	0.00
1.12	**0.63**	0.48	0.48	**0.48**	0.45	0.45
1.13	**0.90**	0.64	0.00	**0.78**	0.74	0.00
1.14	**0.95**	0.68	0.72	**0.82**	0.81	**0.82**

The best performer for the relative item is marked in bold. The asterisk “*” denotes that the program is unable to generate a result under our current computing environment.

**Table 7:** *EC* and *NC* of one-to-one alignment results for *hsa*-*atc*.

*Pathways*	*EC*			*NC*		
	MPBR	CAMPways	SubMAP	MPBR	CAMPways	SubMAP
1.1	0.14	**0.24**	*	0.44	**0.45**	*
1.2	**0.14**	0.00	0.00	**0.12**	0.06	0.06
1.3	**0.81**	0.61	*	**0.65**	0.61	*
1.4	**0.48**	0.33	*	**0.37**	0.35	*
1.5	**0.44**	0.33	*	**0.32**	0.30	*
1.6	**0.74**	0.54	0.44	**0.72**	0.68	0.63
1.7	**0.54**	0.38	0.39	**0.53**	0.47	0.48
1.8	**0.63**	0.55	*	**0.51**	0.46	*
1.9	**0.00**	**0.00**	**0.00**	**0.07**	0.05	**0.07**
1.1	**0.53**	0.51	0.00	**0.59**	0.56	0.00
1.11	**0.33**	0.25	0.00	**0.38**	**0.38**	0.00
1.12	**0.63**	0.46	0.47	**0.49**	0.45	0.45
1.13	**0.88**	0.69	0.67	**0.82**	0.76	0.75
1.14	**0.74**	0.32	0.21	**0.61**	0.57	0.47

The best performer for the relative item is marked in bold.The asterisk “*” denotes that the program is unable to generate a result under our current computing environment.

**Table 8:** *EC* and *NC* of one-to-one alignment results for *mmu*-*atc*.

*Pathways*	*EC*			*NC*		
	MPBR	CAMPways	SubMAP	MPBR	CAMPways	SubMAP
1.1	**0.17**	**0.17**	*	**0.38**	**0.38**	*
1.2	0.14	**0.29**	0.00	**0.11**	0.00	0.06
1.3	**0.80**	0.61	*	**0.63**	0.59	*
1.4	**0.48**	0.33	*	**0.37**	0.35	*
1.5	**0.46**	0.34	*	**0.34**	0.30	*
1.6	**0.76**	0.55	0.46	**0.71**	0.68	0.63
1.7	**0.51**	0.34	0.35	**0.50**	0.44	0.45
1.8	**0.65**	0.59	*	**0.52**	0.49	*
1.9	**0.00**	**0.00**	**0.00**	**0.07**	0.05	**0.07**
1.1	**0.55**	0.54	0.00	**0.59**	0.55	0.00
1.11	**0.33**	0.25	0.00	**0.38**	**0.38**	0.00
1.12	**0.64**	0.47	0.47	**0.49**	0.45	0.45
1.13	**0.87**	0.67	0.66	**0.81**	0.75	0.74
1.14	**0.65**	0.34	0.22	**0.61**	0.58	0.49

The best performer for the relative item is marked in bold. The asterisk “*” denotes that the program is unable to generate a result under our current computing environment.

**Table 9:** *EC* and *NC* of one-to-one alignment results for *mmu*-*eco*.

*Pathways*	*EC*			*NC*		
	MPBR	CAMPways	SubMAP	MPBR	CAMPways	SubMAP
1.1	**0.30**	0.16	*	**0.41**	**0.41**	*
1.2	0.14	**0.29**	0.00	**0.33**	0.17	0.28
1.3	**0.75**	0.47	*	**0.57**	0.53	*
1.4	**0.55**	0.26	*	**0.39**	0.34	*
1.5	**0.48**	0.32	*	**0.29**	0.28	*
1.6	**0.89**	0.68	0.00	**0.82**	0.78	0.00
1.7	**0.40**	0.24	0.21	**0.37**	0.33	0.34
1.8	**0.70**	0.58	*	**0.57**	0.55	*
1.9	**0.00**	**0.00**	**0.00**	**0.08**	0.06	**0.08**
1.1	**0.58**	0.52	0.00	**0.57**	0.55	0.00
1.11	**0.42**	0.33	0.00	**0.36**	**0.36**	0.00
1.12	**0.65**	0.49	0.48	**0.48**	0.45	0.45
1.13	**0.90**	0.67	0.00	**0.78**	0.75	0.00
1.14	**0.94**	0.73	0.69	**0.82**	0.80	0.79

The best performer for the relative item is marked in bold. The asterisk “*” denotes that the program is unable to generate a result under our current computing environment.

**Table 10:** *ELCCS* of one-to-one alignment results for *hsa-eco* and *hsa-atc*.

*Pathways*	*hsa-eco*			*hsa-atc*		
	MPBR	CAMPways	SubMAP	MPBR	CAMPways	SubMAP
1.1	**14**	6	*	**9**	**9**	*
1.2	**5**	4	2	**5**	1	2
1.3	**986**	735	*	**763**	728	*
1.4	**99**	33	*	**54**	20	*
1.5	**459**	57	*	**459**	174	*
1.6	**534**	515	0	**526**	378	392
1.7	**374**	210	175	**374**	264	282
1.8	**57**	**57**	*	**57**	45	*
1.9	**3**	2	0	**3**	1	0
1.10	**155**	134	0	**144**	129	0
1.11	**5**	**5**	0	**4**	3	0
1.12	**2878**	2435	2499	**2878**	2304	2342
1.13	**316**	301	0	**292**	263	275
1.14	**224**	195	215	**224**	103	107

The best performer for the relative item is marked in bold. The asterisk “*” denotes that the program is unable to generate a result under our current computing environment.

**Table 11:** *ELCCS* of one-to-one alignment results for *mmu-atc* and *mmu-eco*.

*Pathways*	*mmu-atc*			*mmu-eco*		
	MPBR	CAMPways	SubMAP	MPBR	CAMPways	SubMAP
1.1	**9**	4	*	**14**	7	*
1.2	**5**	1	2	**5**	2	2
1.3	**746**	725	*	**986**	711	*
1.4	**54**	20	*	**99**	33	*
1.5	**289**	174	*	**289**	162	*
1.6	**508**	376	390	**534**	497	0
1.7	**183**	155	131	**183**	148	150
1.8	**53**	41	*	**53**	**53**	*
1.9	**3**	1	0	**3**	2	0
1.10	**144**	143	0	**155**	134	0
1.11	**4**	3	0	**5**	**5**	0
1.12	**2754**	2324	2330	**2754**	2436	2453
1.13	**294**	258	276	**316**	301	0
1.14	**204**	100	103	**204**	198	198

The best performer for the relative item is marked in bold. The asterisk “*” denotes that the program is unable to generate a result under our current computing environment.

**Table 12:** *C-1tomany* and *CR-1tomany* of one-to-many alignment results for *hsa*-*eco*.

*Pathways*	*C-1tomany*			*CR-1tomany*		
	MPBR	CAMPways	SubMAP	MPBR	CAMPways	SubMAP
1.1	**0**	**0**	*	**0.00**	**0.00**	*
1.2	**0**	**0**	**0**	**0.00**	**0.00**	**0.00**
1.3	**40**	31	*	0.01	**0.48**	*
1.4	**10**	2	*	**0.22**	**0.22**	*
1.5	**1922**	20	*	**0.53**	0.44	*
1.6	**505**	21	*	0.16	**0.53**	*
1.7	**575**	13	8	**0.85**	0.30	0.19
1.8	**55**	2	*	**0.75**	0.14	*
1.9	**0**	**0**	**0**	**0.00**	**0.00**	**0.00**
1.10	**276**	11	*	0.15	**0.23**	*
1.11	**2**	1	0	**1.00**	0.50	0.00
1.12	**15735**	*	*	**0.39**	*	*
1.13	**119**	11	*	0.13	**0.44**	*
1.14	**2781**	7	2	**1.00**	0.54	0.22

The best performer for the relative item is marked in bold. The asterisk “*” denotes that the program is unable to generate a result under our current computing environment.

**Table 13:** *C-1tomany* and *CR-1tomany* of one-to-many alignment results for *hsa*-*atc*.

*Pathways*	*C-1tomany*			*CR-1tomany*		
	MPBR	CAMPways	SubMAP	MPBR	CAMPways	SubMAP
1.1	**0**	**0**	*	**0.00**	**0.00**	*
1.2	**0**	**0**	**0**	**0.00**	**0.00**	**0.00**
1.3	**4956**	30	*	**0.50**	0.46	*
1.4	**10**	1	*	**0.32**	0.17	*
1.5	**1968**	23	*	**0.53**	0.51	*
1.6	**2376**	22	*	**0.93**	0.73	*
1.7	**683**	22	8	**0.87**	0.37	0.21
1.8	**55**	2	*	**0.75**	0.13	*
1.9	**0**	**0**	**0**	**0.00**	**0.00**	**0.00**
1.10	**276**	14	*	0.15	**0.27**	*
.11	**2**	1	1	**1.00**	0.50	0.14
1.12	**13420**	*	*	**0.38**	*	*
1.13	**1679**	13	7	**1.00**	0.59	0.44
1.14	**689**	5	5	**1.00**	0.45	0.36

The best performer for the relative item is marked in bold. The asterisk “*” denotes that the program is unable to generate a result under our current computing environment.

**Table 14:** *C-1tomany* and *CR-1tomany* of one-to-many alignment results for *mmu*-*atc*.

*Pathways*	*C-1tomany*			*CR-1tomany*		
	MPBR	CAMPways	SubMAP	MPBR	CAMPways	SubMAP
1.1	**0**	**0**	*	**0.00**	**0.00**	*
1.2	**0**	**0**	**0**	**0.00**	**0.00**	**0.00**
1.3	**4910**	30	*	**0.50**	0.45	*
1.4	**10**	1	*	**0.32**	0.17	*
1.5	**1956**	24	*	**0.53**	0.52	*
1.6	**2244**	20	*	**0.93**	0.67	*
1.7	**655**	16	5	**0.86**	0.28	0.12
1.8	**58**	2	*	**0.77**	0.14	*
1.9	**0**	**0**	**0**	**0.00**	**0.00**	**0.00**
1.10	**276**	11	*	0.15	**0.24**	*
1.11	**2**	1	1	**1.00**	0.50	0.14
1.12	**13175**	*	*	**0.38**	*	*
1.13	**1679**	14	7	**1.00**	0.64	0.44
1.14	**557**	7	4	**1.00**	0.64	0.29

The best performer for the relative item is marked in bold. The asterisk “*” denotes that the program is unable to generate a result under our current computing environment.

**Table 15:** *C-1tomany* and *CR-1tomany* of one-to-many alignment results for *mmu*-*eco*.

*Pathways*	*C-1tomany*			*CR-1tomany*		
	MPBR	CAMPways	SubMAP	MPBR	CAMPways	SubMAP
1.1	**0**	**0**	*	**0.00**	**0.00**	*
1.2	**0**	**0**	**0**	**0.00**	**0.00**	**0.00**
1.3	**47**	33	*	0.01	**0.45**	*
1.4	**10**	2	*	**0.22**	**0.22**	*
1.5	**1910**	19	*	**0.53**	0.45	*
1.6	**405**	22	*	0.14	**0.54**	*
1.7	**562**	9	7	**0.85**	0.20	0.16
1.8	**64**	1	*	**0.79**	0.07	*
1.9	**0**	**0**	**0**	**0.00**	**0.00**	**0.00**
1.10	**276**	10	*	0.15	**0.20**	*
1.11	**2**	1	0	**1.00**	0.50	0.00
1.12	**15004**	*	*	**0.38**	*	*
1.13	**119**	13	0	0.13	**0.54**	0.00
1.14	**2146**	8	1	**1.00**	0.57	0.13

The best performer for the relative item is marked in bold. The asterisk “*” denotes that the program is unable to generate a result under our current computing environment.

### Same-domain One-to-one Alignments

In this subsection, we discuss the quality of the same-domain one-to-one alignments produced by MPBR and other comparative methods. Tables 1–3 summarize the one-to-one alignment results for the same domain species with respect to distinct performance indices.

As shown in Tables 1–3, over all 28 instances, MPBR has the highest values of *EC*, *NC* and *ELCCS* for 19, 18 and 18 instances respectively, whereas all three methods obtain equal values of *EC*, *NC* and *ELCCS* for 5, 6 and 7 instances respectively. Additionally, MPBR and CAMPways obtain equal values of *EC*, *NC* and *ELCCS* for 4, 4 and 3 instances respectively. These experimental results emphasize that, for the same-domain one-to-one alignment, in most cases, our MPBR method outperforms other comparative methods not only in topological accuracy but also in biological relevance of the results. Thanks to the use of structural similarity among the neighbors of reactions, MPBR is able to improve the topological and biological accuracy of the alignments.

### Same-domain One-to-many Alignments

In this subsection, we compare the quality of the same-domain one-to-many alignments produced by MPBR and other comparative methods. The values of *C-1tomany* and *CR-1tomany* of the one-to-many alignment results for the same domain species are shown in Tables 4 and 5.

From Tables 4 and 5, we can see that, MPBR performs the best with the highest values of *C-1tomany* and *CR-1tomany* in 22 and 15 out of all 28 instances respectively. For 4 instances, all three methods obtained the same values of *C-1tomany* and *CR-1tomany*, while the value of *C-1tomany* of MPBR is lower than CAMPways for 2 instances and is lower than SubMAP for 1 instance. The value of *CR-1tomany* of MPBR is lower than SubMAP for 4 instances, and some values of *CR-1tomany* of MPBR are lower than CAMPways for 7 instances. This means that, in most cases, MPBR is able to return more correct one-to-many reaction mappings than CAMPways and SubMAP in the same-domain one-to-many alignment. On the other hand, when the size of the pathway becomes large, SubMAP is unable to generate a result for 9 instances under the current computing environment while MPBR and CAMPways are not restricted to the size of the pathway in the same-domain one-to-many alignment.

### Across-domains One-to-one Alignments

This subsection discusses the quality of the across-domains one-to-one alignments produced by MPBR and other comparative methods. Tables 6–11 present the one-to-one alignment results for different domain species with respect to distinct performance indices.

As can be seen from Tables 6–11, over all 56 instances, MPBR performs better than the other two methods with the highest values of *EC*, *NC* and *ELCCS* for 47, 42 and 51 instances respectively. Some values of *EC* and *NC* of MPBR are a bit lower than those of CAMPways for 4 and 3 instances respectively. This demonstrates that, in most cases, MPBR is also capable of achieving better topological accuracy and biological relevance of the alignment results than other two comparative methods in across-domains one-to-one alignment.

In addition, from Tables 1–3 and Tables 6–11 we can find that the values of *EC*, *NC* and *ELCCS* of the same-domain alignments are obviously higher than those values of across-domains alignments. This is also consistent with the analysis that the biological relevance of the species within the same domain is much stronger [10]. Thus, we can employ the alignments of metabolic pathways to analyze the evolution of species.

### Across-domains One-to-many Alignments

In this subsection, we compare the quality of the across-domains one-to-many alignments produced by MPBR and other comparative methods. The values of *C-1tomany* and *CR-1tomany* of the one-to-many alignment results for different domain species are shown in Tables 12–15.

From Tables 12–15, we can see that, MPBR achieves the best values of *C-1tomany* and *CR-1tomany* compared to other comparative methods in 44 and 32 out of 56 instances respectively. For only 10 out of 56 instances MPBR fails to be the best. On the other hand, combining Tables 12–15 and S1 Table, we can find that, for the across-domains one-to-many alignment, when the size of the pathway is large enough with thousands of reactions, possibly due to the exhaustive search of reaction sets, CAMPways and SubMAP are unable to generate a result for 34 and 4 instances respectively under the current computing environment. In contrast, for these instances, the values of *C-1tomany* and *CR-1tomany* of MPBR are still high enough without being affected by the size of pathway. While the comparative methods suffer from the size of large-scale pathway, MPBR overcomes this problem and returns more correct one-to-many reaction mappings.

The above analysis of *C-1tomany* and *CR-1tomany* shows that, in most instances, MPBR also performs better than the other two methods in across-domains one-to-many alignment. In conclusion, the results from subsection ‘Same-domain One-to-many Alignments’ and subsection ‘Across-domains One-to-many Alignments’ demonstrate that MPBR is an effective method in retrieving one-to-many reaction mappings in the alignment of metabolic pathways.

### Running time and memory requirements

In the experiments of one-to-one and one-to-many alignments, we have tested a total of 168 instances. In some cases, SubMAP and CAMPWays consumed an unusually long time until running out of memory, Table 16 summaries the percentage of the instances that can be solved by MPBR, CAMPWays and SubMAP respectively, and the average running time for the solved instances. In Table 16, *PSI* represents the percentage of the solved instances of each method, in one-to-one alignment, *ART1* denotes the average running time for the 64 instances solved by all three methods and *ART2* represents the average running time for the 84 instances solved by MPBR and CAMPWays, and in one-to-many alignment, *ART3* denotes the average running time for the 41 instances solved by all three methods, and *ART4* represents the average running time for the 80 instances solved by MPBR and CAMPWays.

**Table 16:** The percentage of the solved instances and the average running time for the solved instances (in seconds).

Methods	One-to-one alignment			One-to-many alignment		
	*PSI*	*ART1*	*ART2*	*PSI*	*ART3*	*ART4*
MPBR	100%(84/84)	693.03	526.21	100%(84/84)	199.65	232.45
CAMPways	100%(84/84)	1595.59	1171.42	95%(80/84)	499.98	518.56
SubMAP	76%(64/84)	15.12	-	49%(41/84)	299.1	-

“-” means that this item is not applicable for SubMAP.

As can be seen from Table 16, for the one-to-one alignment, although the average running time for the 64 solved instances of SubMAP is shorter than CAMPWays and MPBR, SubMAP failed to solve 20 out of 84 instances since it took an unusually long time until running out of memory in these unsolved instances, whereas both CAMPWays and MPBR solved all the instances. Meanwhile we can see that, for the one-to-one alignment, MPBR consumed less time than CAMPWays for the 84 instances solved by MPBR and CAMPWays.

For the one-to-many alignment, we can observe from Table 16 that, MPBR spent less time for the 41 instances solved by all three methods in comparison to CAMPWays and SubMAP; in addition, compared with CAMPWays, MPBR took less time for the 80 solved instances of MPBR and CAMPWays, and MPBR solved all the 84 instances while both CAMPWays and SubMAP did not.

### Many-to-many Alignments

In addition to one-to-many alignments, we can also reveal alternative pathways that have similar functions by finding many-to-many reaction mappings between different pathways. This subsection discusses whether MPBR can accurately find such mappings in the alignment of metabolic pathways. The many-to-many reaction mappings include 2-to-2, 2-to-3 and 3-to-3 reaction mappings. Both CAMPways and SubMAP do not implement the functionality of many-to-many alignment. Table 17 shows the *C-manytomany* of many-to-many alignment results of MPBR.

**Table 17:** *C-manytomany* of many-to-many alignment results of MPBR.

*Pathways*	*Same domain*		*Across domains*			
	*eco-atc*	*hsa-mmu*	*hsa-eco*	*hsa-atc*	*mmu-atc*	*mmu-eco*
1.1	0	2	0	0	0	0
1.2	0	0	0	0	0	0
1.3	23367	18420	19252	16145	16076	19085
1.4	62	59	82	16	16	82
1.5	5320	6271	5247	5323	5302	5221
1.6	5088	6434	8224	4277	4222	7522
1.7	764	1952	589	800	740	542
1.8	10	59	13	19	18	12
1.9	2	0	0	0	0	0
1.10	6063	14523	9628	9628	9628	9628
1.11	0	5	2	0	2	0
1.12	53398	60859	52767	41791	41395	50243
1.13	4115	4348	4177	3335	3335	4177
1.14	358	3733	3573	539	484	1322

Both *Escherichia coli* (*eco*) and *Agrobacterium tumefaciens* (*atc*) are single-cell microorganisms, *Homo sapiens* (*hsa*) and *Mus musculus* (*mmu*) are complex organisms with cell membranes. Table 17 demonstrates that there are a number of many-to-many reaction mappings between the species among the same domain and among different domains. These results suggest that many-to-many reaction mappings frequently appear in nature. MPBR has the capability in finding many-to-many reaction mappings between different pathways to obtain biologically meaningful alignments.

### Case study

**Fig 4:**
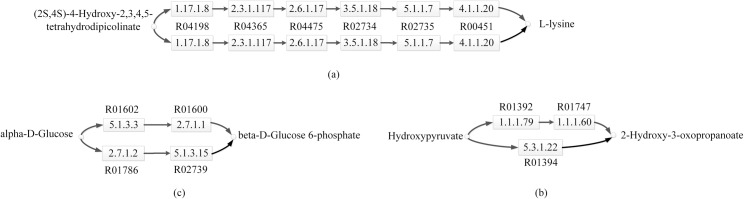
Sample alignments. The upper reactions are a part of the pathways of *atc*, whereas the lower reactions are a part of the pathways of *eco*. Reactions are represented by their KEGG identifiers. Enzymes are shown in EC numbers. The compounds are depicted by small circles. (a) One-to-one reaction mappings extracted from the alignment of the metabolic pathways of lysine biosynthesis in *atc* and *eco*. (b) A one-to-many reaction mapping extracted from the alignment of the metabolic pathways of Glyoxylate and dicarboxylate metabolism in *atc* and *eco*. (c) A many-to-many reaction mapping extracted from the alignment of the metabolic pathways of Glycolysis in *atc* and *eco*.

In this subsection, we present three cases (as shown in Fig 4) to discuss how to comparatively analyze metabolic pathways using MPBR, CAMPways and SubMAP. We represent the reactions by their KEGG identifiers. First, we used MPBR, CAMPways and SubMAP to perform one-to-one alignment for the metabolic pathways of lysine biosynthesis in *atc* and *eco*. The result is shown in Fig 4(A). Lysine biosynthesis pathway consists of 6 enzymes arranged in a linear topology, transforming the substrate (2S,4S)-4-Hydroxy- 2,3,4,5-tetrahydrodipicolinate into L-lysine. We observed that the pathways of lysine biosynthesis are identical between *atc* and *eco*. This implies a common ancestral pathway, which is consistent with the theory that pathways for synthesis of proteinogenic amino acids were established before ancient organisms diverged into archaea, bacteria, and eucarya [28].

On the other hand, one-to-many or many-to-many reaction mappings in the alignment of pathways may uncover additional interesting evolutionary phenomena, or alternative pathways performing the same or similar function. An example is a one-to-many reaction mapping in Fig 4(B). MPBR obtains this mapping by performing one-to-many alignment for the metabolic pathways of Glyoxylate and dicarboxylate metabolism in *atc* and *eco*. Both CAMPways and SubMAP fail to find this mapping in this alignment. In Fig 4(B), the *eco* reaction R01394 [Hydroxypyruvate < = > 2-Hydroxy-3-oxopropanoate] was mapped to the *atc* reactions R01392 [D-Glycerate + NADP+ < = > Hydroxypyruvate + NADPH + H+] and R01747 [D-Glycerate + NADP+ < = > 2-Hydroxy-3-oxopropanoate + NADPH + H+]. Since both R01394 and R01392 share one input compound Hydroxypyruvate and both R01394 and R01747 share one output compound 2-Hydroxy-3-oxopropanoate, the reaction R01394 in *eco*, catalyzed by 5.3.1.22, is functionally similar to the succession of the two reactions R01392 and R01747 in *atc*, catalyzed by 1.1.1.79 and 1.1.1.60. Biologically, this indicates that the functionality of 5.3.1.22 in *eco* is analogous to the combined functionality of the two enzymes 1.1.1.79 and 1.1.1.60 in *atc*. This may imply an intriguing case of either gene fusion in *eco* or gene duplication in *atc*. This needs to be further investigated to reveal the biological scene that leads to this event; nevertheless, it provides an elicitation in this direction.

Another example is a many-to-many reaction mapping in Fig 4(C). MPBR obtains this mapping by performing many-to-many alignment for the metabolic pathways of Glycolysis in *atc* and *eco*. Both CAMPways and SubMAP do not implement the functionality of many-to-many alignment. In Fig 4(C), MPBR mapped the *atc* reactions R01602 [alpha-D-Glucose < = > beta-D-Glucose] and R01600 [ATP + beta-D-Glucose < = > ADP + beta-D-Glucose 6-phosphate] to the *eco* reactions R01786 [ATP + alpha-D-Glucose < = > ADP + alpha-D-Glucose 6-phosphate] and R02739 [alpha-D-Glucose 6-phosphate < = > beta-D-Glucose 6-phosphate]. As can be seen from Fig 4(C), alpha-D-Glucose can be transformed into beta-D-Glucose 6-phosphate through reactions R01602 and R01600 in *atc*, while this transformation can be done through reactions R01786 and R02739 in *eco*. That is, by allowing many-to-many reaction mappings in the alignments, MPBR has successfully found different alternative pathways that have similar function through different sets of reactions.

## Conclusions

In this paper, we have proposed an alignment method MPBR for finding reaction mappings between two metabolic pathways. We have formalized the connected relation between reactions as binary relation of reactions, and have shown how to employ the multiplications of zero-one matrices for binary relation of reactions to search reaction sets in a small number of steps to uncover one-to-many and many-to-many reaction mappings between two metabolic pathways. This provides the first step in the process of exploiting the relation between reactions in the alignment of metabolic pathways. The success of MPBR is primarily due to the use of the multiplications of zero-one matrices for binary relation of reactions in finding reaction sets, which avoids the exhaustive search for reaction sets and increases the accuracy of the alignments of the alternative pathways. Furthermore, we introduce a measure of topological similarity of reactions, which compares the structural similarity of the *k*-neighborhood subgraphs of the reactions, and employ this similarity metric to improve the accuracy of the alignments.

In most cases, MPBR obtains alignment results with higher values of *EC*, *NC*, *ELCCS*, *C-1tomany* and *CR-1tomany* than CAMPways and SubMAP, and accurately returns more biologically relevant mappings. Moreover, our method also provides a user-defined parameter for finding many-to-many reaction mappings in the alignments, while both CAMPways and SubMAP do not support many-to-many alignment. Thus, MPBR enriches the means of one-to-many and/or many-to-many alignments of metabolic pathways.

In order to further improve biological relevance of resulting mappings, one feasible solution is to use context-specific information content, such as semantic similarity of the gene ontology (GO) terms or sequence information, to compute homological similarity of reactions. Another interesting issue is to exploit binary relation of reactions to identify functional motifs in metabolic pathways.

## Supporting Information

S1 Table The number of nodes and the number of edges of the pathways. (DOC)

S2 Table *NC* of one-to-one alignment results for the fourth level of the FGC hierarchy. (DOC)

S3 Table *NC* of one-to-one alignment results for the third level of the FGC hierarchy. (DOC)

S4 Table *NC* of one-to-one alignment results for the second level of the FGC hierarchy. (DOC)

S5 Table *NC* of one-to-one alignment results for the first level of the FGC hierarchy. (DOC)
