# Supplementary material for: Aligning Metabolic Pathways Exploiting Binary Relation of Reactions
Source: PLoS One. 2016 Dec 9;11(12):e0168044. doi: 10.1371/journal.pone.0168044 (PMC5148114; doi:10.1371/journal.pone.0168044)
Supplement: S1 Table — (DOC) [file pone.0168044.s001.doc]

**S1 Table. The number of nodes and the number of edges of the pathways.**

| *Pathways* | *Organisms* | *Number of Nodes* | *Number of Edges* | *Pathways* | *Organisms* | *Number of Nodes* | *Number of Edges* |
| --- | --- | --- | --- | --- | --- | --- | --- |
| 1.1 | *hsa* | 107 | 63 | 1.8 | *hsa* | 69 | 88 |
|  | *mmu* | 100 | 63 |  | *mmu* | 65 | 83 |
|  | *eco* | 67 | 15 |  | *eco* | 59 | 79 |
|  | *atc* | 108 | 34 |  | *atc* | 49 | 59 |
| 1.2 | *hsa* | 17 | 7 | 1.9 | *hsa* | 84 | 3 |
|  | *mmu* | 18 | 7 |  | *mmu* | 84 | 3 |
|  | *eco* | 13 | 5 |  | *eco* | 41 | 30 |
|  | *atc* | 10 | 6 |  | *atc* | 32 | 21 |
| 1.3 | *hsa* | 220 | 792 | 1.10 | *hsa* | 154 | 388 |
|  | *mmu* | 217 | 771 |  | *mmu* | 153 | 379 |
|  | *eco* | 250 | 1011 |  | *eco* | 170 | 296 |
|  | *atc* | 208 | 748 |  | *atc* | 164 | 279 |
| 1.4 | *hsa* | 31 | 36 | 1.11 | *hsa* | 17 | 6 |
|  | *mmu* | 31 | 36 |  | *mmu* | 14 | 6 |
|  | *eco* | 62 | 110 |  | *eco* | 22 | 12 |
|  | *atc* | 54 | 63 |  | *atc* | 29 | 12 |
| 1.5 | *hsa* | 382 | 614 | 1.12 | *hsa* | 1520 | 3316 |
|  | *mmu* | 363 | 575 |  | *mmu* | 1466 | 3166 |
|  | *eco* | 127 | 232 |  | *eco* | 1127 | 3083 |
|  | *atc* | 137 | 231 |  | *atc* | 1104 | 2545 |
| 1.6 | *hsa* | 160 | 530 | 1.13 | *hsa* | 88 | 296 |
|  | *mmu* | 160 | 512 |  | *mmu* | 89 | 298 |
|  | *eco* | 166 | 536 |  | *eco* | 97 | 326 |
|  | *atc* | 130 | 359 |  | *atc* | 83 | 249 |
| 1.7 | *hsa* | 255 | 413 | 1.14 | *hsa* | 72 | 224 |
|  | *mmu* | 241 | 389 |  | *mmu* | 71 | 204 |
|  | *eco* | 171 | 236 |  | *eco* | 69 | 191 |
|  | *atc* | 198 | 282 |  | *atc* | 47 | 92 |
