# Supplementary material for: Aligning Metabolic Pathways Exploiting Binary Relation of Reactions
Source: PLoS One. 2016 Dec 9;11(12):e0168044. doi: 10.1371/journal.pone.0168044 (PMC5148114; doi:10.1371/journal.pone.0168044)
Supplement: S2 Table — (DOC) [file pone.0168044.s002.doc]

**S2 Table. *NC* of one-to-one alignment results for the fourth level of the FGC hierarchy.** The asterisk denotes that the program cannot generate a result under our current computing environment.

**S2 Table (a). *NC* of one-to-one alignment results for *eco*-*atc***.

| *Pathways* | | *NC* | | |
| --- | --- | --- | --- | --- |
|  |  | MPAL | CAMPways | SubMAP |
| *eco*-1.1 | *atc*-1.1 | 0.5277778 | 0.5462963 | 0 |
| *eco*-1.2 | *atc*-1.2 | 0.6923077 | 0.6923077 | 0.6923077 |
| *eco*-1.3 | *atc*-1.3 | 0.748 | 0.684 | 0.68 |
| *eco*-1.4 | *atc*-1.4 | 0.7903226 | 0.7741935 | 0.7741935 |
| *eco*-1.5 | *atc*-1.5 | 0.7810219 | 0.7664233 | 0 |
| *eco*-1.6 | *atc*-1.6 | 0.7951807 | 0.7590361 | 0.7349398 |
| *eco*-1.7 | *atc*-1.7 | 0.7272728 | 0.7121212 | 0 |
| *eco*-1.8 | *atc*-1.8 | 0.6949152 | 0.6271187 | 0.6440678 |
| *eco*-1.9 | *atc*-1.9 | 0.8048781 | 0.7073171 | 0.7560976 |
| eco-1.10 | *atc*-1.10 | 0.8411765 | 0.8117647 | 0.817647 |
| *eco*-1.11 | *atc*-1.11 | 0.5862069 | 0.6206896 | 0 |
| *eco*-1.12 | *atc*-1.12 | 0.7994676 | 0.757764 | 0.7613132 |
| *eco*-1.13 | *atc*-1.13 | 0.8659794 | 0.8350515 | 0.8350515 |
| *eco*-1.14 | *atc*-1.14 | 0.7101449 | 0.6521739 | 0.5797101 |

**S2 Table (b). *NC* of one-to-one alignment results for *hsa*-*mmu*.**

| *Pathways* | | *NC* | | |
| --- | --- | --- | --- | --- |
|  |  | MPAL | CAMPways | SubMAP |
| *hsa*-1.1 | *mmu*-1.1 | 0.9345794 | 0.9345794 | 0.9345794 |
| *hsa*-1.2 | *mmu*-1.2 | 0.9444444 | 0.9444444 | 0 |
| *hsa*-1.3 | *mmu*-1.3 | 0.9909091 | 0.9681818 | 0.9772728 |
| *hsa*-1.4 | *mmu*-1.4 | 1 | 1 | 1 |
| *hsa*-1.5 | *mmu*-1.5 | 0.9450262 | 0.9188482 | 0.9319372 |
| *hsa*-1.6 | *mmu*-1.6 | 0.99375 | 0.99375 | 0.61875 |
| *hsa*-1.7 | *mmu*-1.7 | 0.945098 | 0.9294118 | 0.9294118 |
| *hsa*-1.8 | *mmu*-1.8 | 0.942029 | 0.9275363 | 0.9275363 |
| *hsa*-1.9 | *mmu*-1.9 | 1 | 0.6785714 | 0.8928571 |
| *hsa*-1.10 | *mmu*-1.10 | 0.9805195 | 0.9675325 | 0.9805195 |
| *hsa*-1.11 | *mmu*-1.11 | 0.8235294 | 0.8235294 | 0.8235294 |
| *hsa*-1.12 | *mmu*-1.12 | 0.9631579 | 0.9217106 | 0.9440789 |
| *hsa*-1.13 | *mmu*-1.13 | 0.9887641 | 0.9887641 | 0 |
| *hsa*-1.14 | *mmu*-1.14 | 1 | 0.9861111 | 0.9722222 |

**S2 Table (c). *NC* of one-to-one alignment results for *hsa*-*eco*.**

| *Pathways* | | *NC* | | |
| --- | --- | --- | --- | --- |
|  |  | MPAL | CAMPways | SubMAP |
| *hsa*-1.1 | *eco*-1.1 | 0.4579439 | 0.4579439 | * |
| *hsa*-1.2 | *eco*-1.2 | 0.3529412 | 0.1764706 | 0.2941177 |
| *hsa*-1.3 | *eco*-1.3 | 0.632 | 0.624 | * |
| *hsa*-1.4 | *eco*-1.4 | 0.4193548 | 0.3870968 | * |
| *hsa*-1.5 | *eco*-1.5 | 0.3089005 | 0.2801047 | * |
| *hsa*-1.6 | *eco*-1.6 | 0.813253 | 0.7831326 | 0 |
| *hsa*-1.7 | *eco*-1.7 | 0.427451 | 0.3882353 | 0.4 |
| *hsa*-1.8 | *eco*-1.8 | 0.6231884 | 0.6086956 | * |
| *hsa*-1.9 | *eco*-1.9 | 0.0833333 | 0.0714286 | 0.0952381 |
| *hsa*-1.10 | eco-1.10 | 0.5823529 | 0.5764706 | 0 |
| *hsa*-1.11 | *eco*-1.11 | 0.3636364 | 0.3636364 | 0 |
| *hsa*-1.12 | *eco*-1.12 | 0.525 | 0.4953947 | 0.5 |
| *hsa*-1.13 | *eco*-1.13 | 0.7835051 | 0.742268 | 0 |
| *hsa*-1.14 | *eco*-1.14 | 0.8194444 | 0.8055556 | 0.8194444 |

**S2 Table (d). *NC* of one-to-one alignment results for *hsa*-*atc***

| *Pathways* | | *NC* | | |
| --- | --- | --- | --- | --- |
|  |  | MPAL | CAMPways | SubMAP |
| *hsa*-1.1 | *atc*-1.1 | 0.4722222 | 0.462963 | * |
| *hsa*-1.2 | *atc*-1.2 | 0.1176471 | 0.0588235 | 0.0588235 |
| *hsa*-1.3 | *atc*-1.3 | 0.6954545 | 0.6727273 | * |
| *hsa*-1.4 | *atc*-1.4 | 0.4074074 | 0.3703704 | * |
| *hsa*-1.5 | *atc*-1.5 | 0.3769634 | 0.3115183 | * |
| *hsa*-1.6 | *atc*-1.6 | 0.71875 | 0.675 | 0.625 |
| *hsa*-1.7 | *atc*-1.7 | 0.5529412 | 0.4862745 | 0.5019608 |
| *hsa*-1.8 | *atc*-1.8 | 0.5797101 | 0.5362319 | * |
| *hsa*-1.9 | *atc*-1.9 | 0.0714286 | 0.0595238 | 0.0952381 |
| *hsa*-1.10 | *atc*-1.10 | 0.597561 | 0.597561 | 0 |
| *hsa*-1.11 | *atc*-1.11 | 0.5172414 | 0.3793103 | 0 |
| *hsa*-1.12 | *atc*-1.12 | 0.5342105 | 0.4980263 | 0.4881579 |
| *hsa*-1.13 | *atc*-1.13 | 0.8181818 | 0.7613636 | 0.75 |
| *hsa*-1.14 | *atc*-1.14 | 0.6111111 | 0.5694444 | 0.4722222 |

**S2 Table (e). *NC* of one-to-one alignment results for *mmu*-*atc***

| *Pathways* | | *NC* | | |
| --- | --- | --- | --- | --- |
|  |  | MPAL | CAMPways | SubMAP |
| *mmu*-1.1 | *atc*-1.1 | 0.4074074 | 0.3888889 | * |
| *mmu*-1.2 | *atc*-1.2 | 0.1111111 | 0 | 0.0555556 |
| *mmu*-1.3 | *atc*-1.3 | 0.6820276 | 0.6774194 | * |
| *mmu*-1.4 | *atc*-1.4 | 0.4074074 | 0.3703704 | * |
| *mmu*-1.5 | *atc*-1.5 | 0.3994491 | 0.3168044 | * |
| *mmu*-1.6 | *atc*-1.6 | 0.7125 | 0.675 | 0.63125 |
| *mmu*-1.7 | *atc*-1.7 | 0.5228216 | 0.4647303 | 0.4813278 |
| *mmu*-1.8 | *atc*-1.8 | 0.6 | 0.5538462 | * |
| *mmu*-1.9 | *atc*-1.9 | 0.0714286 | 0.0595238 | 0.0952381 |
| *mmu*-1.10 | *atc*-1.10 | 0.6036586 | 0.5792683 | 0 |
| *mmu*-1.11 | *atc*-1.11 | 0.5862069 | 0.4137931 | 0 |
| *mmu*-1.12 | *atc*-1.12 | 0.5327421 | 0.4965894 | 0.4890859 |
| *mmu*-1.13 | *atc*-1.13 | 0.8089888 | 0.752809 | 0.741573 |
| *mmu*-1.14 | *atc*-1.14 | 0.6056338 | 0.5774648 | 0.4929577 |

**S2 Table (f). *NC* of one-to-one alignment results for *mmu*-*eco***

| *Pathways* | | *NC* | | |
| --- | --- | --- | --- | --- |
|  |  | MPAL | CAMPways | SubMAP |
| *mmu*-1.1 | *eco*-1.1 | 0.42 | 0.41 | * |
| *mmu*-1.2 | *eco*-1.2 | 0.3333333 | 0.1666667 | 0.2777778 |
| *mmu*-1.3 | *eco*-1.3 | 0.64 | 0.624 | * |
| *mmu*-1.4 | *eco*-1.4 | 0.4193548 | 0.3870968 | * |
| *mmu*-1.5 | *eco*-1.5 | 0.3443526 | 0.2975207 | * |
| *mmu*-1.6 | *eco*-1.6 | 0.8192771 | 0.7771084 | 0 |
| *mmu*-1.7 | *eco*-1.7 | 0.4024896 | 0.3609959 | 0.3817428 |
| *mmu*-1.8 | *eco*-1.8 | 0.6153846 | 0.5783132 | * |
| *mmu*-1.9 | *eco*-1.9 | 0.0833333 | 0.0714286 | 0.0952381 |
| *mmu*-1.10 | eco-1.10 | 0.5941176 | 0.5705882 | 0 |
| *mmu*-1.11 | *eco*-1.11 | 0.3636364 | 0.3636364 | 0 |
| *mmu*-1.12 | *eco*-1.12 | 0.5245566 | 0.5006821 | 0.5006821 |
| *mmu*-1.13 | *eco*-1.13 | 0.7835051 | 0.7525773 | 0 |
| *mmu*-1.14 | *eco*-1.14 | 0.8169014 | 0.8028169 | 0.7887324 |
