# Supplementary material for: Aligning Metabolic Pathways Exploiting Binary Relation of Reactions
Source: PLoS One. 2016 Dec 9;11(12):e0168044. doi: 10.1371/journal.pone.0168044 (PMC5148114; doi:10.1371/journal.pone.0168044)
Supplement: S3 Table — (DOC) [file pone.0168044.s003.doc]

**S3 Table. *NC* of one-to-one alignment results for the third level of the FGC hierarchy.** The asterisk denotes that the program cannot generate a result under our current computing environment.

**S3 Table (a). *NC* of one-to-one alignment results for *eco*-*atc***

| *Pathways* | | *NC* | | |
| --- | --- | --- | --- | --- |
|  |  | MPAL | CAMPways | SubMAP |
| *eco*-1.1 | *atc*-1.1 | 0.5277778 | 0.5462963 | 0 |
| *eco*-1.2 | *atc*-1.2 | 0.6923077 | 0.7692308 | 0.7692308 |
| *eco*-1.3 | *atc*-1.3 | 0.776 | 0.708 | 0.7 |
| *eco*-1.4 | *atc*-1.4 | 0.8064516 | 0.7741935 | 0.7903226 |
| *eco*-1.5 | *atc*-1.5 | 0.8029197 | 0.7737227 | 0 |
| *eco*-1.6 | *atc*-1.6 | 0.8012048 | 0.7590361 | 0.7349398 |
| *eco*-1.7 | *atc*-1.7 | 0.7575758 | 0.7272728 | 0 |
| *eco*-1.8 | *atc*-1.8 | 0.6949152 | 0.6271187 | 0.6610169 |
| *eco*-1.9 | *atc*-1.9 | 0.8292683 | 0.7073171 | 0.7560976 |
| eco-1.10 | *atc*-1.10 | 0.8529412 | 0.817647 | 0.817647 |
| *eco*-1.11 | *atc*-1.11 | 0.7931035 | 0.6551724 | 0 |
| *eco*-1.12 | *atc*-1.12 | 0.8198758 | 0.7781721 | 0.7817214 |
| *eco*-1.13 | *atc*-1.13 | 0.8762887 | 0.8350515 | 0.8350515 |
| *eco*-1.14 | *atc*-1.14 | 0.7101449 | 0.6521739 | 0.5797101 |

**S3 Table (b). *NC* of one-to-one alignment results for *hsa*-*mmu***

| *Pathways* | | *NC* | | |
| --- | --- | --- | --- | --- |
|  |  | MPAL | CAMPways | SubMAP |
| *hsa*-1.1 | *mmu*-1.1 | 0.9345794 | 0.9345794 | 0.9345794 |
| *hsa*-1.2 | *mmu*-1.2 | 0.9444444 | 0.9444444 | 0 |
| *hsa*-1.3 | *mmu*-1.3 | 0.9909091 | 0.9727273 | 0.9818182 |
| *hsa*-1.4 | *mmu*-1.4 | 1 | 1 | 1 |
| *hsa*-1.5 | *mmu*-1.5 | 0.960733 | 0.9267016 | 0.9397906 |
| *hsa*-1.6 | *mmu*-1.6 | 0.99375 | 0.99375 | 0.61875 |
| *hsa*-1.7 | *mmu*-1.7 | 0.9529412 | 0.9333333 | 0.9333333 |
| *hsa*-1.8 | *mmu*-1.8 | 0.9565218 | 0.9275363 | 0.9275363 |
| *hsa*-1.9 | *mmu*-1.9 | 1 | 0.6785714 | 0.8928571 |
| *hsa*-1.10 | *mmu*-1.10 | 0.9805195 | 0.9675325 | 0.9805195 |
| *hsa*-1.11 | *mmu*-1.11 | 0.8235294 | 0.8235294 | 0.8235294 |
| *hsa*-1.12 | *mmu*-1.12 | 0.9743421 | 0.9256579 | 0.9480263 |
| *hsa*-1.13 | *mmu*-1.13 | 0.9887641 | 0.9887641 | 0 |
| *hsa*-1.14 | *mmu*-1.14 | 1 | 0.9861111 | 0.9722222 |

**S3 Table (c). *NC* of one-to-one alignment results for *hsa*-*eco***

| *Pathways* | | *NC* | | |
| --- | --- | --- | --- | --- |
|  |  | MPAL | CAMPways | SubMAP |
| *hsa*-1.1 | *eco*-1.1 | 0.4859813 | 0.4672897 | * |
| *hsa*-1.2 | *eco*-1.2 | 0.3529412 | 0.1764706 | 0.3529412 |
| *hsa*-1.3 | *eco*-1.3 | 0.676 | 0.656 | * |
| *hsa*-1.4 | *eco*-1.4 | 0.4354839 | 0.3870968 | * |
| *hsa*-1.5 | *eco*-1.5 | 0.3638744 | 0.2827225 | * |
| *hsa*-1.6 | *eco*-1.6 | 0.8253012 | 0.7831326 | 0 |
| *hsa*-1.7 | *eco*-1.7 | 0.4627451 | 0.4117647 | 0.4078431 |
| *hsa*-1.8 | *eco*-1.8 | 0.6956522 | 0.6231884 | * |
| *hsa*-1.9 | *eco*-1.9 | 0.1071429 | 0.0714286 | 0.0952381 |
| *hsa*-1.10 | eco-1.10 | 0.6117647 | 0.5941176 | 0 |
| *hsa*-1.11 | *eco*-1.11 | 0.4090909 | 0.4545455 | 0 |
| *hsa*-1.12 | *eco*-1.12 | 0.5644737 | 0.5236842 | 0.525 |
| *hsa*-1.13 | *eco*-1.13 | 0.7835051 | 0.742268 | 0 |
| *hsa*-1.14 | *eco*-1.14 | 0.8194444 | 0.8055556 | 0.8194444 |

**S3 Table (d). *NC* of one-to-one alignment results for *hsa*-*atc***

| *Pathways* | | *NC* | | |
| --- | --- | --- | --- | --- |
|  |  | MPAL | CAMPways | SubMAP |
| *hsa*-1.1 | *atc*-1.1 | 0.5092593 | 0.4907408 | * |
| *hsa*-1.2 | *atc*-1.2 | 0.1176471 | 0.1176471 | 0.1764706 |
| *hsa*-1.3 | *atc*-1.3 | 0.6954545 | 0.7090909 | * |
| *hsa*-1.4 | *atc*-1.4 | 0.4259259 | 0.3703704 | * |
| *hsa*-1.5 | *atc*-1.5 | 0.4371728 | 0.3167539 | * |
| *hsa*-1.6 | *atc*-1.6 | 0.73125 | 0.675 | 0.625 |
| *hsa*-1.7 | *atc*-1.7 | 0.5843138 | 0.5058824 | 0.5176471 |
| *hsa*-1.8 | *atc*-1.8 | 0.6086956 | 0.5507246 | * |
| *hsa*-1.9 | *atc*-1.9 | 0.0714286 | 0.0595238 | 0.0952381 |
| *hsa*-1.10 | *atc*-1.10 | 0.6158537 | 0.6280488 | 0 |
| *hsa*-1.11 | *atc*-1.11 | 0.5517241 | 0.3793103 | 0 |
| *hsa*-1.12 | *atc*-1.12 | 0.5855263 | 0.5269737 | 0.5177631 |
| *hsa*-1.13 | *atc*-1.13 | 0.8295454 | 0.7613636 | 0.75 |
| *hsa*-1.14 | *atc*-1.14 | 0.6111111 | 0.5694444 | 0.4722222 |

**S3 Table (e). *NC* of one-to-one alignment results for *mmu*-*atc***

| *Pathways* | | *NC* | | |
| --- | --- | --- | --- | --- |
|  |  | MPAL | CAMPways | SubMAP |
| *mmu*-1.1 | *atc*-1.1 | 0.4444445 | 0.4259259 | * |
| *mmu*-1.2 | *atc*-1.2 | 0.1111111 | 0 | 0.1666667 |
| *mmu*-1.3 | *atc*-1.3 | 0.6820276 | 0.7142857 | * |
| *mmu*-1.4 | *atc*-1.4 | 0.4259259 | 0.3703704 | * |
| *mmu*-1.5 | *atc*-1.5 | 0.4269972 | 0.3250689 | * |
| *mmu*-1.6 | *atc*-1.6 | 0.725 | 0.675 | 0.63125 |
| *mmu*-1.7 | *atc*-1.7 | 0.5518673 | 0.4937759 | 0.5103735 |
| *mmu*-1.8 | *atc*-1.8 | 0.6307693 | 0.5538462 | * |
| *mmu*-1.9 | *atc*-1.9 | 0.0714286 | 0.0595238 | 0.0952381 |
| *mmu*-1.10 | *atc*-1.10 | 0.6280488 | 0.5853658 | 0 |
| *mmu*-1.11 | *atc*-1.11 | 0.5862069 | 0.4137931 | 0 |
| *mmu*-1.12 | *atc*-1.12 | 0.5716234 | 0.5279673 | 0.5218281 |
| *mmu*-1.13 | *atc*-1.13 | 0.8202247 | 0.752809 | 0.741573 |
| *mmu*-1.14 | *atc*-1.14 | 0.6056338 | 0.5774648 | 0.4929577 |

**S3 Table (f). *NC* of one-to-one alignment results for *mmu*-*eco***

| *Pathways* | | *NC* | | |
| --- | --- | --- | --- | --- |
|  |  | MPAL | CAMPways | SubMAP |
| *mmu*-1.1 | *eco*-1.1 | 0.45 | 0.45 | * |
| *mmu*-1.2 | *eco*-1.2 | 0.3333333 | 0.1666667 | 0.3333333 |
| *mmu*-1.3 | *eco*-1.3 | 0.676 | 0.66 | * |
| *mmu*-1.4 | *eco*-1.4 | 0.4354839 | 0.3870968 | * |
| *mmu*-1.5 | *eco*-1.5 | 0.3746556 | 0.3030303 | * |
| *mmu*-1.6 | *eco*-1.6 | 0.8313253 | 0.7771084 | 0 |
| *mmu*-1.7 | *eco*-1.7 | 0.439834 | 0.3817428 | 0.3941909 |
| *mmu*-1.8 | *eco*-1.8 | 0.6923077 | 0.5846154 | * |
| *mmu*-1.9 | *eco*-1.9 | 0.1071429 | 0.0714286 | 0.0952381 |
| *mmu*-1.10 | eco-1.10 | 0.6235294 | 0.5823529 | 0 |
| *mmu*-1.11 | *eco*-1.11 | 0.3636364 | 0.4545455 | 0 |
| *mmu*-1.12 | *eco*-1.12 | 0.5579809 | 0.5225102 | 0.5300136 |
| *mmu*-1.13 | *eco*-1.13 | 0.7835051 | 0.7525773 | 0 |
| *mmu*-1.14 | *eco*-1.14 | 0.8169014 | 0.8028169 | 0.7887324 |
