# Supplementary material for: Aligning Metabolic Pathways Exploiting Binary Relation of Reactions
Source: PLoS One. 2016 Dec 9;11(12):e0168044. doi: 10.1371/journal.pone.0168044 (PMC5148114; doi:10.1371/journal.pone.0168044)
Supplement: S5 Table — (DOC) [file pone.0168044.s005.doc]

**S5 Table. *NC* of one-to-one alignment results for the first level of the FGC hierarchy.** The asterisk denotes that the program cannot generate a result under our current computing environment.

**S5 Table (a). *NC* of one-to-one alignment results for *eco*-*atc***

| *Pathways* | | *NC* | | |
| --- | --- | --- | --- | --- |
|  |  | MPAL | CAMPways | SubMAP |
| *eco*-1.1 | *atc*-1.1 | 0.75 | 0.5740741 | 0 |
| *eco*-1.2 | *atc*-1.2 | 0.6923077 | 0.7692308 | 0.7692308 |
| *eco*-1.3 | *atc*-1.3 | 0.8 | 0.724 | 0.732 |
| *eco*-1.4 | *atc*-1.4 | 0.8709678 | 0.7903226 | 0.8064516 |
| *eco*-1.5 | *atc*-1.5 | 0.8248175 | 0.7737227 | 0 |
| *eco*-1.6 | *atc*-1.6 | 0.8072289 | 0.7590361 | 0.7349398 |
| *eco*-1.7 | *atc*-1.7 | 0.8080808 | 0.7828283 | 0 |
| *eco*-1.8 | *atc*-1.8 | 0.7966102 | 0.6779661 | 0.7118644 |
| *eco*-1.9 | *atc*-1.9 | 0.8536586 | 0.7073171 | 0.7560976 |
| eco-1.10 | *atc*-1.10 | 0.8647059 | 0.8529412 | 0.8294118 |
| *eco*-1.11 | *atc*-1.11 | 0.862069 | 0.6896552 | 0 |
| *eco*-1.12 | *atc*-1.12 | 0.8482698 | 0.8198758 | 0.8198758 |
| *eco*-1.13 | *atc*-1.13 | 0.8865979 | 0.8350515 | 0.8350515 |
| *eco*-1.14 | *atc*-1.14 | 0.7246377 | 0.6521739 | 0.5797101 |

**S5 Table (b). *NC* of one-to-one alignment results for *hsa*-*mmu***

| *Pathways* | | *NC* | | |
| --- | --- | --- | --- | --- |
|  |  | MPAL | CAMPways | SubMAP |
| *hsa*-1.1 | *mmu*-1.1 | 0.9345794 | 0.9345794 | 0.9345794 |
| *hsa*-1.2 | *mmu*-1.2 | 1 | 0.9444444 | 0 |
| *hsa*-1.3 | *mmu*-1.3 | 0.9909091 | 0.9727273 | 0.9818182 |
| *hsa*-1.4 | *mmu*-1.4 | 1 | 1 | 1 |
| *hsa*-1.5 | *mmu*-1.5 | 0.9764398 | 0.9319372 | 0.9397906 |
| *hsa*-1.6 | *mmu*-1.6 | 0.99375 | 0.99375 | 0.6375 |
| *hsa*-1.7 | *mmu*-1.7 | 0.9607843 | 0.9333333 | 0.9333333 |
| *hsa*-1.8 | *mmu*-1.8 | 0.9710145 | 0.9275363 | 0.9275363 |
| *hsa*-1.9 | *mmu*-1.9 | 1 | 0.6785714 | 0.8928571 |
| *hsa*-1.10 | *mmu*-1.10 | 0.9805195 | 0.9675325 | 0.9805195 |
| *hsa*-1.11 | *mmu*-1.11 | 1 | 0.8235294 | 0.8235294 |
| *hsa*-1.12 | *mmu*-1.12 | 0.9756579 | 0.9282895 | 0.9480263 |
| *hsa*-1.13 | *mmu*-1.13 | 0.9887641 | 0.9887641 | 0 |
| *hsa*-1.14 | *mmu*-1.14 | 1 | 0.9861111 | 0.9861111 |

**S5 Table (c). *NC* of one-to-one alignment results for *hsa*-*eco***

| *Pathways* | | *NC* | | |
| --- | --- | --- | --- | --- |
|  |  | MPAL | CAMPways | SubMAP |
| *hsa*-1.1 | *eco*-1.1 | 0.6542056 | 0.5514019 | * |
| *hsa*-1.2 | *eco*-1.2 | 0.4117647 | 0.1764706 | 0.3529412 |
| *hsa*-1.3 | *eco*-1.3 | 0.724 | 0.68 | * |
| *hsa*-1.4 | *eco*-1.4 | 0.5645161 | 0.3870968 | * |
| *hsa*-1.5 | *eco*-1.5 | 0.4633508 | 0.2931937 | * |
| *hsa*-1.6 | *eco*-1.6 | 0.8253012 | 0.7891566 | 0 |
| *hsa*-1.7 | *eco*-1.7 | 0.5960785 | 0.509804 | 0.5058824 |
| *hsa*-1.8 | *eco*-1.8 | 0.7971014 | 0.6811594 | * |
| *hsa*-1.9 | *eco*-1.9 | 0.1071429 | 0.0714286 | 0.1071429 |
| *hsa*-1.10 | eco-1.10 | 0.6411765 | 0.6529412 | 0 |
| *hsa*-1.11 | *eco*-1.11 | 0.4545455 | 0.5909091 | 0 |
| *hsa*-1.12 | *eco*-1.12 | 0.6361842 | 0.5684211 | 0.5644737 |
| *hsa*-1.13 | *eco*-1.13 | 0.7938144 | 0.7525773 | 0 |
| *hsa*-1.14 | *eco*-1.14 | 0.8194444 | 0.8055556 | 0.8194444 |

**S5 Table (d). *NC* of one-to-one alignment results for *hsa*-*atc***

| *Pathways* | | *NC* | | |
| --- | --- | --- | --- | --- |
|  |  | MPAL | CAMPways | SubMAP |
| *hsa*-1.1 | *atc*-1.1 | 0.7962963 | 0.75 | * |
| *hsa*-1.2 | *atc*-1.2 | 0.1176471 | 0.1176471 | 0.1764706 |
| *hsa*-1.3 | *atc*-1.3 | 0.7272728 | 0.75 | * |
| *hsa*-1.4 | *atc*-1.4 | 0.5555556 | 0.3888889 | * |
| *hsa*-1.5 | *atc*-1.5 | 0.539267 | 0.3219895 | * |
| *hsa*-1.6 | *atc*-1.6 | 0.73125 | 0.68125 | 0.63125 |
| *hsa*-1.7 | *atc*-1.7 | 0.6862745 | 0.627451 | 0.6196079 |
| *hsa*-1.8 | *atc*-1.8 | 0.6811594 | 0.5797101 | * |
| *hsa*-1.9 | *atc*-1.9 | 0.0714286 | 0.0595238 | 0.0952381 |
| *hsa*-1.10 | *atc*-1.10 | 0.6585366 | 0.6951219 | 0 |
| *hsa*-1.11 | *atc*-1.11 | 0.6896552 | 0.4827586 | 0 |
| *hsa*-1.12 | *atc*-1.12 | 0.6743421 | 0.5789474 | 0.5657895 |
| *hsa*-1.13 | *atc*-1.13 | 0.8295454 | 0.7727273 | 0.7613636 |
| *hsa*-1.14 | *atc*-1.14 | 0.6111111 | 0.5694444 | 0.4722222 |

**S5 Table (e). *NC* of one-to-one alignment results for** ***mmu*-*atc***

| *Pathways* | | *NC* | | |
| --- | --- | --- | --- | --- |
|  |  | MPAL | CAMPways | SubMAP |
| *mmu*-1.1 | *atc*-1.1 | 0.7314815 | 0.6944444 | * |
| *mmu*-1.2 | *atc*-1.2 | 0.1111111 | 0 | 0.1666667 |
| *mmu*-1.3 | *atc*-1.3 | 0.718894 | 0.7511521 | * |
| *mmu*-1.4 | *atc*-1.4 | 0.5555556 | 0.3888889 | * |
| *mmu*-1.5 | *atc*-1.5 | 0.5289256 | 0.338843 | * |
| *mmu*-1.6 | *atc*-1.6 | 0.725 | 0.68125 | 0.6375 |
| *mmu*-1.7 | *atc*-1.7 | 0.6721992 | 0.6141079 | 0.6182573 |
| *mmu*-1.8 | *atc*-1.8 | 0.7076923 | 0.5692308 | * |
| *mmu*-1.9 | *atc*-1.9 | 0.0714286 | 0.0595238 | 0.0952381 |
| *mmu*-1.10 | *atc*-1.10 | 0.6585366 | 0.6402439 | 0 |
| *mmu*-1.11 | *atc*-1.11 | 0.6896552 | 0.4137931 | 0 |
| *mmu*-1.12 | *atc*-1.12 | 0.6637108 | 0.5770805 | 0.5675307 |
| *mmu*-1.13 | *atc*-1.13 | 0.8202247 | 0.7640449 | 0.752809 |
| *mmu*-1.14 | *atc*-1.14 | 0.6056338 | 0.5774648 | 0.4929577 |

**S5 Table (f). *NC* of one-to-one alignment results for *mmu*-*eco***

| *Pathways* | | *NC* | | |
| --- | --- | --- | --- | --- |
|  |  | MPAL | CAMPways | SubMAP |
| *mmu*-1.1 | *eco*-1.1 | 0.63 | 0.54 | * |
| *mmu*-1.2 | *eco*-1.2 | 0.3888889 | 0.2222222 | 0.3333333 |
| *mmu*-1.3 | *eco*-1.3 | 0.728 | 0.684 | * |
| *mmu*-1.4 | *eco*-1.4 | 0.5645161 | 0.3870968 | * |
| *mmu*-1.5 | *eco*-1.5 | 0.4793389 | 0.3057851 | * |
| *mmu*-1.6 | *eco*-1.6 | 0.8313253 | 0.7831326 | 0 |
| *mmu*-1.7 | *eco*-1.7 | 0.5767635 | 0.4937759 | 0.5145229 |
| *mmu*-1.8 | *eco*-1.8 | 0.8 | 0.6307693 | * |
| *mmu*-1.9 | *eco*-1.9 | 0.1071429 | 0.0714286 | 0.1071429 |
| *mmu*-1.10 | eco-1.10 | 0.6411765 | 0.6470588 | 0 |
| *mmu*-1.11 | *eco*-1.11 | 0.4545455 | 0.5 | 0 |
| *mmu*-1.12 | *eco*-1.12 | 0.6302865 | 0.5668486 | 0.5648022 |
| *mmu*-1.13 | *eco*-1.13 | 0.7938144 | 0.7628866 | 0 |
| *mmu*-1.14 | *eco*-1.14 | 0.8169014 | 0.8028169 | 0.7887324 |
